# Ribbons of Light: Emerging (Sb,Bi)(S,Se)(Br,I) Van der Waals Chalcohalides for Next‐Generation Energy Applications

**DOI:** 10.1002/smll.202505430

**Published:** 2025-07-23

**Authors:** Ivan Caño, Alejandro Navarro‐Güell, Edoardo Maggi, Axel Gon Medaille, David Rovira, Alex Jimenez‐Arguijo, Oriol Segura, Arnau Torrens, Maykel Jimenez, Cibrán López, Pol Benítez, Claudi Cazorla, Zac Jehl, Yuancai Gong, José‐Miguel Asensi, Lorenzo Calvo‐Barrio, Lluís Soler, Jordi Llorca, Josep‐Lluís Tamarit, Beatriz Galiana, Mirjana Dimitrievska, Nazaret Ruiz‐Marín, Hao Zhe Chun, Lydia Wong, Joaquim Puigdollers, Marcel Placidi, Edgardo Saucedo

**Affiliations:** ^1^ Photovoltaic Lab – Micro and Nano Technologies Group (MNT) Electronic Engineering Department Universitat Politècnica de Catalunya (UPC) EEBE, Av Eduard Maristany 10–14 Barcelona 08019 Spain; ^2^ Barcelona Centre for Multiscale Science and Engineering Universitat Politècnica de Catalunya (UPC) Av Eduard Maristany 10–14 Barcelona 08019 Spain; ^3^ Group of Characterization of Materials (GCM) Physics Department Universitat Politècnica de Catalunya (UPC) EEBE, Av Eduard Maristany 10–14 Barcelona 08019 Spain; ^4^ Nanoengineering of Materials Applied to Energy (NEMEN) Chemical Engineering Department Institute of Energy Technologies Universitat Politècnica de Catalunya (UPC) EEBE, Av Eduard Maristany 10–14 Barcelona 08019 Spain; ^5^ Applied Physics Department Universitat de Barcelona (UB) C. Martí I Franquès 1 Barcelona 08028 Spain; ^6^ Scientific and Technological Centers (CCiTUB) Universitat de Barcelona (UB) C. Lluís Solé i Sabaris 1–3 Barcelona 08028 Spain; ^7^ Electronics and Biomedical Engineering Department Universitat de Barcelona (UB) In2UB, C. Martí i Franqupes 1 Barcelona 08028 Spain; ^8^ Physics Department Universidad Carlos III de Madrid Av. Universidad 40 Leganés 28911 Spain; ^9^ Nanomaterials Spectroscopy and Imaging group Swiss Federal Laboratories for Materials Science and Technology (EMPA) Überlandstrasse 129 Dübendorf 8600 Switzerland; ^10^ Departamento de Máquinas y Motores Térmicos Universidad de Cádiz Campus Universitario de Puerto Real s/n. 11510 Puerto Real, Cádiz Cádiz 11510 Spain; ^11^ School of Materials Science and Engineering Nanyang Technological University Singapore 639798 Singapore

**Keywords:** anisotropic materials, chalcohalides, photoelectrocatalysis, photovoltaics, Van der Waals

## Abstract

(Sb,Bi)(S,Se)(Br,I) pnictogen chalcohalides constitute an emerging family of Van der Waals (VdW) semiconductors with remarkable potential for energy‐related applications, including photovoltaics (PV), photocatalysis (PC), and photoelectrocatalysis (PEC). These ternary compounds exhibit a quasi‐1D orthorhombic crystalline phase, and an electronic structure analogous to lead‐halide perovskites, making them promising candidates for sustainable and high‐performance energy devices. This study introduces a new versatile and adaptable synthesis methodology, which combines co‐evaporation of binary chalcogenides with reactive annealing under high‐pressure halide atmospheres, to fabricate the eight (Sb,Bi)(S,Se)(Br,I) chalcohalides. Comprehensive structural, compositional, and optoelectronic analyses reveal a wide bandgap range (1.2–2.2 eV), high absorption coefficients, and anisotropic properties driven by unique ribbon‐like morphology. Theoretical and experimental results highlight their high stability, versatile chemical adaptability, and defect‐tolerant characteristics. Moreover, the distinct differences in morphology and crystallization between Sb and Bi‐based compounds, as well as the influence of chalcogen and halogen elements on the optical and structural properties are discussed. Demonstrations of functional devices, including photocatalytic systems, underscore the practical viability of these materials. This work establishes a foundation for the development of pnictogen chalcohalides as scalable and eco‐friendly alternatives for advanced energy applications.

## Graphical abstract

This work presents the first unified synthesis and analysis of the full (Sb,Bi)(S,Se)(Br,I)chalcohalide family. It explores their structural, electronic, andoptoelectronic properties, highlighting anisotropic behaviours, and demonstratingpotential in photovoltaics (PV) and photocatalysis (PC), paving the way foreco‐friendly energy applications.

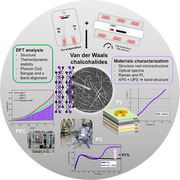

## 1 Introduction

Crystalline and multi‐crystalline silicon, amorphous silicon, CdTe, Cu(In,Ga)(S,Se)_2_, organic polymers, and hybrid lead halide perovskites, are already in, or in track, to the fast‐growing photovoltaic market.^[^ ^1^ ^]^ All these technologies have demonstrated conversion efficiencies in the 20–27% range, and have been considered the vanguard of the photovoltaic materials family.^[^ ^2^ ^]^ However, they face some challenges and limitations, including the presence of scarce or toxic elements (In, Ga, and Te), conflict minerals, problems with instability and degradation (lead halide perovskites, organic solar cells), or low adaptability for customized photovoltaic product design (c‐Si).^[^ ^3^, ^4^, ^5^ ^]^ Furthermore, the continuous penetration of PV into emerging non‐conventional niche markets has led to the necessity of developing materials and devices involving specific characteristics adapted to these emerging applications, such as stability, sustainability, low toxicity and low processing temperatures, as well as functional properties including thickness and bandgap tuneability, and compatibility with flexible and transparent substrates. Indeed, in the current world order of a steadily increasing energy consumption, along with the need to move toward a more sustainable paradigm by reducing carbon emissions and limiting the usage of non‐renewable resources, emerging PV offer innovative solutions for the integration of solar cells into the urban fabric of cities and human environments (PV windows, agrivoltaics), wearables, and indoor PV for powering the fast‐growing internet‐of‐things environment.

In this context, emerging inorganic chalcogenides are experiencing very fast growth. In particular, kesterite solar cells – Cu_2_ZnSn(S,Se)_4_ – have been consistently highlighted as an attractive photovoltaic system, as it is solely constituted by earth‐abundant and non‐toxic components. Additionally, Sb_2_(S,Se)_3_ and heavy pnictogen chalcohalides, have shown great promise owing to their remarkable versatility in terms of processing and intrinsic properties.^[^ ^6^, ^7^ ^]^ Among these newcomer technologies, a family of materials known as VdW semiconductors is rapidly rising out of the plethora of innovative semiconductors that exhibit exotic properties. Overall, VdW compounds can be defined as those that involve strong covalent bonding along one or several directions of their crystalline structure, while exhibiting weak VdW interactions in the other directions. This is the case of layered materials such as boron nitride, which presents strong in‐plane covalent bonds and weaker interlayer interactions, as well as mono‐layered compounds like graphene or silicene. These characteristics typically lead to anisotropic properties, such as enhanced thermal conductivity and higher carrier mobility along the planes, rather than perpendicular to them.^[^ ^8^, ^9^ ^]^

**Figure 1:**
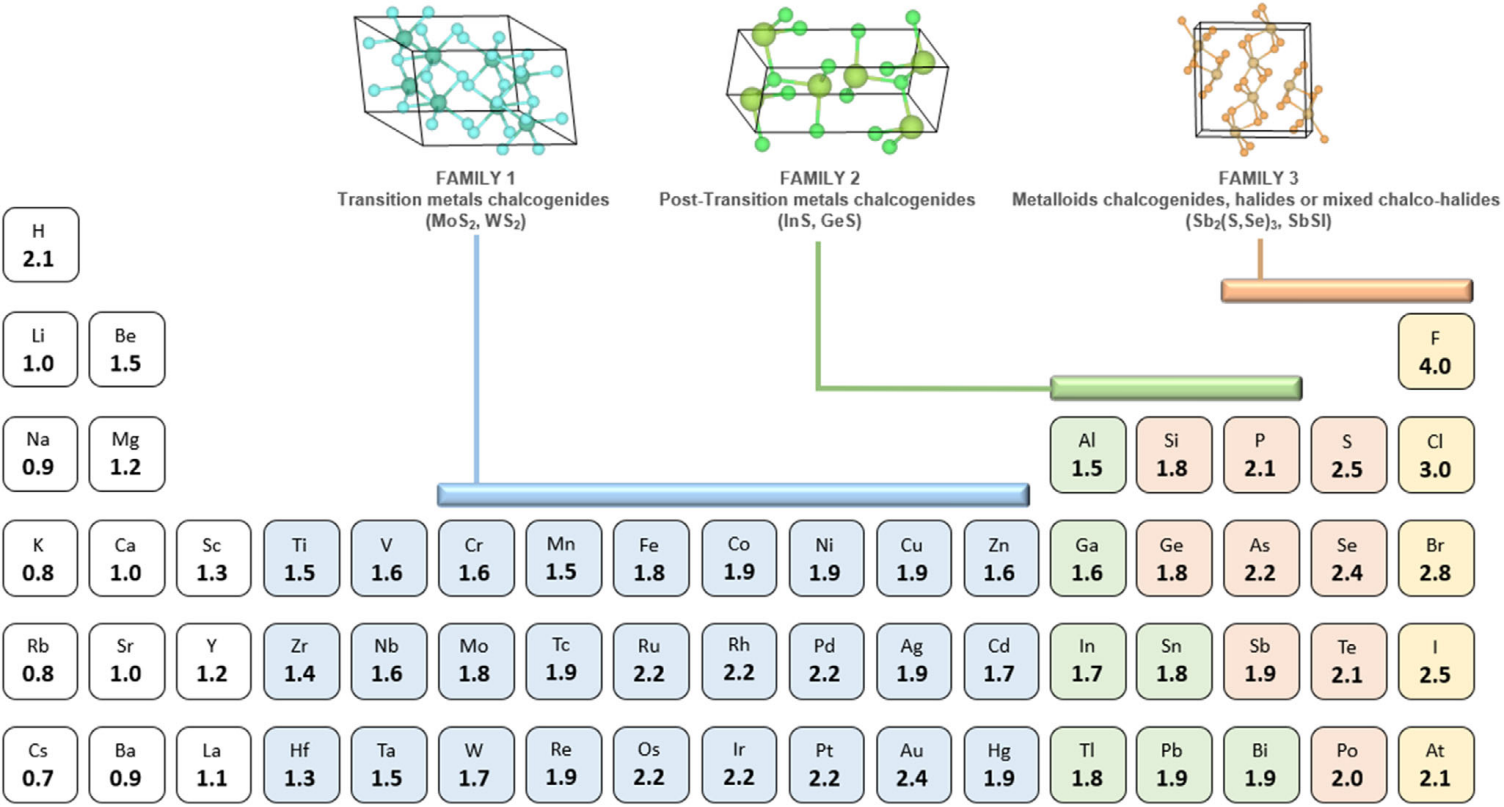
Schematic representation of selected elements from the Periodic Table with the corresponding electronegativity, highlighting the three most relevant families of vdW compounds. On top, the structure of representative materials for the different families is shown, including MoS_2_ for Family 1, InS for Family 2, and Sb_2_Se_3_ for Family 3.

VdW materials are not limited to these 2D compounds. Conveniently, there is a simple empirical rule which allows to discriminate whether a material can exhibit in‐plane or inter‐ribbon VdW bonding, resulting in a low‐dimensional structure: that is, the difference between the electronegativities of anions and cations has to be lower than 1.5 in order to exhibit the VdW characteristics.^[^ ^10^ ^]^ Importantly, as this is a purely phenomenological rule, it might not be universally accurate, however, it offers a good framework for predicting potential candidates. **Figure** **1** shows a schematic representation of the periodic table, indicating different families of materials which result from combining chalcogen and/or halogen anions, with metals and semimetals that possess relatively high electronegativities, so that Δ*χ* < 1.5. These materials include the family of transition metal chalcogenides (e.g., MoS_2_, MoSe_2_, and WS_2_ – see “Family 1” in Figure 1), which present a layered structure similar to graphene, and have gained substantial attention for implementation into electrochemical, photocatalytic, and optoelectronic systems.^[^ ^11^ ^]^ The second family is constituted by post‐transition metal chalcogenides (including In, Ge, Sn, and Pb – “Family 2” in Figure 1), which are nevertheless difficult to obtain in pure form due to the possibility of these metals to exhibit intermediate oxidation states (e.g., Sn^2+^ and Sn^4+^).^[^ ^12^ ^]^

Finally, materials belonging to the third group (“Family 3” in Figure 1) are often referred to as VdW pnictogen chalcogenides or chalcohalides, and have historically been led by Sb‐based materials. For instance, Sb_2_Se_3_ and Sb_2_(S,Se)_3_ have demonstrated solar cell performance with conversion efficiencies above 10% in a relatively short period of time.^[^ ^13^, ^14^ ^]^ The possibility to tune the morphology from thin films to quasi‐1D ribbons, bandgap from 1.2 up to 1.8 eV approximately, and their good adaptation toward substrate and superstrate device configurations make them extremely attractive for different energy applications. In addition, chemical adaptability and customizability of properties can be largely increased by substituting one chalcogen (oxidation state −2) from the structure by two halogens (oxidation state −1), evolving from the Ch‐M‐Ch‐M‐Ch sequence (where Ch is the chalcogen and M the pnictogen), corresponding to a general formula M_2_Ch_3_, toward a Ch‐M‐X‐X‐M‐Ch sequence (X corresponds to halogen), or more compactly as M‐Ch‐X, which corresponds to the general formula MChX. Hence, considering 2 pnictogens (Sb and Bi), 2 chalcogens (S and Se), and 2 halogens (Br and I), up to 8 different ternary VdW compounds can be developed, namely: SbSBr, SbSI, SbSeBr, SbSeI, BiSBr, BiSI, BiSeBr, and BiSeI. Nonetheless, among all these compounds, only a few of them have been successfully synthesized in thin film, and even fewer have been tested for specific technological applications such as PV or PEC. **Table** **1** shows selected experimental and theoretical publications, with relevant parameters and applications reported for this family of ternary emerging chalcohalides.

**Table 1:** Summary of relevant theoretical (highlighted in grey), and experimental results reported in the literature for the eight ternary compounds corresponding to the (Sb,Bi)(S,Se)(Br,I) system.

¿	Exp./Theo.	Synthesis	Structure	Morphology	*E*_g_ [eV]	Other properties	Applications	Ref.
SbSeI	Theo.	–	Orthorhombic, *Pnma* or *Pna2_1_*	–	1.256	–	–	[15, 16]
	Exp.	Coevaporation of Sb_2_Se_3_ and annealing	Orthorhombic, *Pnma*	Nanorods	1.74	High crystalline quality and uniform nanorods	PV devices, Eff. = 0.3%. Micro‐devices with photoactivity	[17]
		SbI_3_ solution spin‐coated onto FTO/BL/mp‐TiO_2_/Sb_2_Se_3_	Orthorhombic, *Pnma*	Thin films	1.67	Excellent uniformity and long‐term stability	PV devices, Eff. up to 4.1%	[18]
SbSeBr	Theo.	–	Orthorhombic *Pnma*	–	1.5	Energy band structure	–	[16]
	Exp.	Coevaporation of Sb_2_Se_3_ and annealing	Orthorhombic, *Pnma*	Nanorods	N.R.	–	PV devices, Eff. = 0.6%	[17]
SbSI	Theo	–	Orthorhombic *Pnma* or *Pna2_1_*	–	1.44– 1.7	Energy band structure	–	[15, 16]
	Exp.	Spin coating DMSO	N.R.	N.R.	2.15	Wavelength dependent photovoltaic effect	PV	[19]
		CBD Sb_2_S_3_ + spin coating DMF SbI_3_	Orthorhombic	SbSI into mp‐TiO_2_	2.15	–	PV Eff. = 3.05%	[20]
		Hydrotherm. Sb_2_S_3_ + spin coating CS_2_ NMP or DMF SbI_3_	Orthorhombic	Nanorods	N.R.	–	PEC photoanode Jpha = 2.5 mA cm^−2^	[21]
SbSBr	Theo	–	Orthorhombic *Pnma* or *Pna2_1_*	Bulk and 1D	1.69–2.06	Thermal stability, good electron mobility	Suggested for transistors, optoelectronics	[22]
BiSeI	Theo.	–	Orthorhombic	–	1.3 2.67	Predicted very high e‐ mobility Band positions	–	[16, 23]
	Exp.	Liquid phase method	Orthorhombic	Nanosheets	1.15	2D structures demonstrated	Photodetector	[24]
		Solid‐state synthesis with Bi_2_Se_3_ and BiI_3_ precursors	Orthorhombic *Pnma*	Needles	1.17	Complex defect and band structure by PL	–	[25]
BiSeBr	Theo.	–	Orthorhombic	–	1.3 2.73	Predicted good h+ and e‐ mobilities Band positions	–	[16, 23]
BiSI	Theo.	–	Orthorhombic *Pnma*	–	1.5	Energy band structure	–	[16]
	Exp.	Spin coating the molecular precursor	Orthorhombic *Pnma*	Flake‐shaped grains	1.57	PL decay lifetimes: 90 ps and 1.03 ¡ns	PV, Eff. = 1.32%	[26]
		Spin coating of BiI3, conversion on BiOI, annealing under H2S to convert into BiSI	Orthorhombic *Pnma*	Flake‐shaped grains	1.57	–	Photodetector with responsivity of 62.1 AW−1 at 10 V	[27]
BiSBr	Theo.	–	Orthorhombic *Pnma*	–	1.8–2.3	–	–	[16, 28]
	Exp.	Spin coating DMSO	Orthorhombic *Pnma*	Nanorods, nanoparticles	1.7–1.91	Lifetimes > 1 ns High IPCE	PV, electrodes	[29]

**Figure 2:**
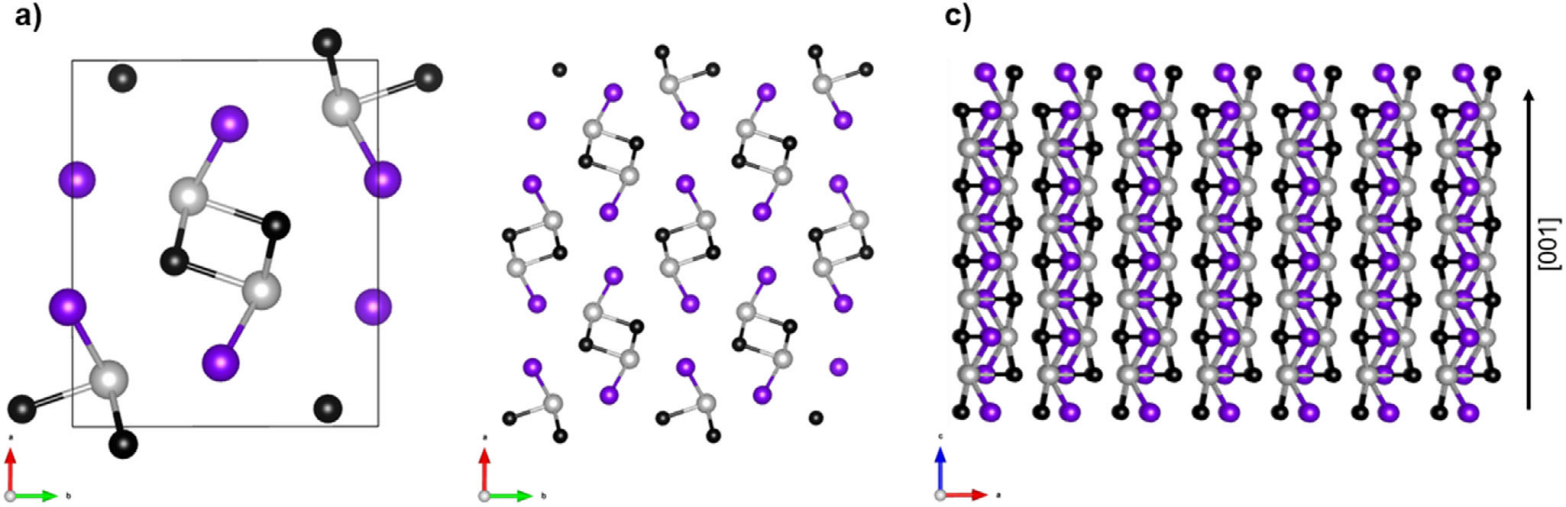
Crystal structure of SbSeI – Sb (black), Se (grey), I (purple) a) Unit cell of the *Pnma* orthorhombic SbSeI. b) SbSeI structure constituted by (SbSeI)_n_ ribbons stacked in parallel along the [001] direction. Note that the atoms at the edge of the ribbons ([100] and [010] directions) are saturated, indicating that when the material is correctly oriented along the *c‐*axis, grain boundaries will be terminated by benign surfaces which do not introduce recombination losses.

The MChX's structure consists of covalently‐bonded ribbons along a single crystallographic direction (i.e., *c*‐axis/[001] direction, see **Figure** **2**), while they are held together by weak VdW interactions in the other directions ([100] and [010]). This structure is equivalent to that of other better‐known quasi‐1D materials, such as Sb_2_Se_3_ and Sb_2_S_3_, which also present an orthorhombic crystal structure belonging to the *Pnma* space group, see Figure 2. In these cases, it has been demonstrated that textured films where the preferred orientation is in the [001] direction (i.e., covalent chains perpendicular to substrate), enhances the optoelectronic properties of the semiconductor, as charge carriers travel more efficiently along the covalently bonded ribbons; suggesting that the transport properties of MChX could also be improved by correctly orienting the material.^[^ ^30^ ^]^ Theoretical analyses have shown that the unique quasi‐1D characteristics of VdW chalcogenides originate from the pnictogen *s^2^* lone pair, which distorts its coordination environment resulting in an asymmetric electronic density. In turn, the direction of the lone pair has a direct effect on the inter‐ribbon binding energies (which are lower under the VdW regime), and the electron/hole effective masses, which show clear anisotropy, with lower values in the *c*‐axis spanning across the covalent chains.^[^ ^31^ ^]^

In addition, MChX compounds have also been appointed as “perovskite‐inspired materials”. This term has been recently coined to describe any group of semiconductors presenting an electronic structure similar to lead‐halide perovskites, regardless of their crystalline structure. The growing interest on developing “perovskite‐inspired materials” arises from state‐of‐the‐art research suggesting that the remarkable defect tolerance properties shown by lead‐halide perovskites originate from their electronic structure, which is characterized by the antibonding nature of the valence band, and the bonding nature of the conduction band. Therefore, there is considerable interest in discovering materials which display analogous band structures and electronic properties, with the anticipation that such compounds will exhibit similar defect tolerance features. In particular, it has been demonstrated that materials involving s^2^ lone electron pairs, such as those constituted by partially oxidized cations like Sb^+3^ or Bi^+3^, exhibit the characteristic antibonding nature of the valence band which is also observed in lead‐halide perovskites.^[^ ^32^, ^33^, ^34^ ^]^ This applies to a number of binary chalcogenides (Bi_2_S_3_, Sb_2_Se_3_) and pnictogen chalcohalides (SbSeI, SbSeBr, SbSI, BiSI…), involving cations with the electronic configuration ns^2^ np⁰ (*n* = 5 for Sb, 6 for Bi). Hence, they have been designated as “perovskite‐inspired materials by electronic analogy”.^[^ ^35^, ^36^ ^]^ However, the role of these electronic features to defect formation and activation energies is still controversial and requires further validation and systematic investigation through both experimental and theoretical approaches.

In summary, chalcohalide materials exhibit many attractive properties for the development of advanced PV applications, such as VdW crystalline structure, electronic structure analogous to perovskites, and sustainable nature (low toxicity, possibility of synthesis through environmentally‐friendly low‐temperature methodologies). However, they have been scarcely investigated, and despite being first fabricated as crystals in the 1960s, the first solar cell was only reported in 2018. Several chalcohalides have yet to be synthesized in thin‐film form, and only some have been tested for optoelectronic applications. Most significantly, SbSeI and SbSI have demonstrated successful implementation into solar cells, achieving efficiencies up to 4.1% and 3%, using an innovative structure based on chalcohalide's deposition onto meso‐porous TiO_2_.^[^ ^18^, ^20^, ^37^ ^]^ Indeed, SbSI is the better‐known of these materials, also showing promise in photoelectrochemistry,^[^ ^21^ ^]^ PC,^[^ ^38^, ^39^ ^]^ sensors,^[^ ^40^, ^41^, ^42^ ^]^ transistors,^[^ ^43^ ^]^ supercapacitors,^[^ ^44^ ^]^ and smart wearables.^[^ ^45^ ^]^ On the other hand, bromides have been very little investigated, with most research focused on simulations and DFT analysis.^[^ ^16^ ^]^ See Table 1 for the most relevant recent publications and benchmarks on Sb/Bi chalcohalides.

Overall, in the literature, there is consensus regarding the orthorhombic structure of all these compounds, with either *Pnma* or *Pna2_1_* as the most probable space groups. On the other hand, bandgaps have been reported in the 1–2 eV range, but there is no consensus regarding the actual value for most of the compounds. In addition, some chalcohalides have been fabricated in quasi‐1D structures, although different procedures have been explored, leading to non‐contrastable properties (Table 1). In addition, theoretical analyses have demonstrated the potential of Sb and Bi chalcohalides to develop compatible properties for several energy applications, such as optimal bandgap for PV or optical detection, and appropriate band positions for PC and PEC. As a highly versatile system in terms of chemical adaptability, the possibility of tuning optoelectronic properties through the synthesis of solid solutions has been suggested as well.

In this work, we present the first “universal method” which allows to synthesize the complete group of ternary (Sb,Bi)(S,Se)(Br,I) VdW compounds based on a single systematic procedure. This method consists on co‐evaporation of the chalcogenide precursors (Sb_2_Se_3_, Sb_2_S_3,_ Bi_2_Se_3_, and Bi_2_S_3_) followed by an innovative reactive thermal annealing under selective halogen atmosphere to convert the precursor into a ternary chalcohalide. A complete analysis of fundamental properties is presented, including structure, composition, morphology, optics, electronic, and transport properties. Thence, for the first time, the eight MChX compounds are synthesized with contrastable properties, and possible applications of selected compounds are demonstrated as well. Remarkably, we present the first successful attempt to fabricate a functional photocatalytic device based on VdW mixed chalcohalide materials.

The results clearly show the high potential of this emerging family of VdW materials for different technological applications, from energy generation and storage to sensors and electronic components, opening new perspectives for implementing VdW materials with low‐dimensional morphologies into advanced technological applications. We would like to highlight that, although the article touches on some recent advancements, showcasing the increasing potential of VdW chalcohalides, the findings presented are predominantly original and have not been previously disclosed or published. These include an extensive characterization work, the coordinated synthesis of the eight compounds, and their implementation into PC and PEC prototypes.

## 2 DFT Modelling Analysis

Recently, the optoelectronic properties of several Sb and Bi chalcohalide materials have been theoretically characterized by means of first‐principles methods based on Density Functional Theory (DFT).^[^ ^16^, ^46^, ^47^, ^48^ ^]^ In this work, we extend this complete and systematic computational DFT analysis of the structural, vibrational, thermodynamic, optoelectronic, and band properties of the eight compounds conforming the family of chalcohalide materials. Comparisons between theoretical and experimental results are also provided.

The DFT results presented in this section were obtained using the Perdew–Burke–Ernzerhof functional revised for solids as the baseline, see a description of the procedure in Methods.^[^ ^49^ ^]^ Given the importance of long‐range dispersion interactions in these materials, the VdW correction approach was employed for the geometry optimizations.^[^ ^50^ ^]^ In addition, spin‐orbit coupling effects (SOC), which can be particularly relevant for Bi‐containing compounds, were also taken into consideration in the ensuing DFT calculations. For the estimation of optoelectronic properties (e.g., band gaps and optical absorption coefficients), the range‐separated hybrid functional HSE06 [HSE06+SOC] was used to minimize the well‐known shortcomings of semi‐local functionals (such as self‐interaction energy errors).^[^ ^51^, ^52^ ^]^

The eight MChX materials have been found to be thermodynamically and vibrationally stable in the orthorhombic phase (space group *Pnma*), see Figure S1 (Supporting Information). This crystalline structure is characterized by 1D ribbons held together by weak VdW interactions, closely resembling the Sb_2_Se_3_ structure.^[^ ^53^ ^]^ The DFT geometry optimizations performed here resulted in crystal symmetries and lattice parameters that are in excellent agreement with the experimental results. For instance, DFT calculations for SbSeI yielded the following values: *a*   =  4.10 Å, *b*  =  8.51 Å and *c*  =  10.27 Å, which are in excellent agreement with the corresponding experimental results: *a*  =  4.15 Å, *b*  =  8.69 Å and *c*  =  10.39 Å (lattice vectors for all parent compounds are summarized in Table S1, Supporting Information). The theoretical lattice parameters were estimated at zero‐temperature conditions, whilst the experimental values have been obtained at room temperature. Therefore, the systematic ≈3% underestimation of the experimental lattice parameters is likely originated by thermal lattice expansion effects.

**Figure 3:**
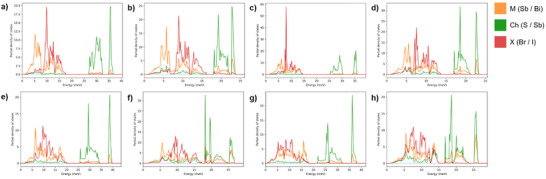
Vibrational phonon partial densities of states of the eight chalcohalides: a) BiSBr, b) BiSeBr, c) BiSI, d) BiSeI, e) SbSBr, f) SbSeBr, g) SbSI, h) SbSeI.

The vibrational stability of all parent chalcohalides in the orthorhombic *Pnma* phase has been studied by means of phonon calculations, see Figure S2a (Supporting Information) for the vibrational phonon spectra of the eight chalcohalides. In all cases, real and positively defined phonon frequencies were ascertained along high symmetry k‐paths in the reciprocal space, confirming their vibrational stability. Interestingly, large phonon bandgaps have appeared for S‐based compounds, while these gaps are almost negligible in Se‐based chalcohalides, see **Figure** **3**. Also, we note that the vibrational phonon spectra at low (0  <  ℏω  <  7 meV), medium (7  <  ℏω  <  17 meV). and high (17  <  ℏω  <  30 meV) energy ranges are essentially dominated by the pnictogen, halogen, and chalcogen elements, respectively. Such a dependency of the phonon energy ranges on atomic species can be understood in terms of their masses, given that lighter atoms typically vibrate at higher frequencies, while heavier atoms vibrate at lower frequencies. Importantly, major differences regarding these band structures can be attributed to the chalcogen, which displaces the position of the most energetic bands. In particular, due to their quasi‐1D nature, sizable phonon bandgaps appear in S‐containing compounds at energies ≈16 meV (see Figure 3), while these are practically absent in Se‐containing compounds.

Notable agreement between theoretical DFT results and Raman spectroscopy experiments is obtained. For instance, for the particular case of SbSeI, experimental active Raman peaks {93, 113, 135, 165, 179, 206} cm^−1^ are in very good agreement with the corresponding DFT phonon frequencies {96, 113, 135, 174, 178, 208} cm^−1^ (see experimental Raman analysis in Section 3). All phonon frequencies and the contribution of each species to the corresponding vibration are listed in Table S2 (Supporting Information). It is noted that the phonon spectrum of SbSI in the centrosymmetric Pnma phase exhibits some imaginary phonon frequencies (Figure 3a). This is due to the fact that at low temperatures, SbSI is ferroelectric, and the Pnma phase becomes stabilized by thermal effects near room temperature.^[^ ^54^ ^]^

Interestingly, a second orthorhombic *Pnma* phase was found to be energetically competitive with respect to the orthorhombic ground state shown in Figure 2. This new structure (see Figure S3, Supporting Information) results from the dislocation of a (001) plane by half a unit cell in the ground‐state *Pnma* phase. In the particular case of SbSI, this secondary *Pnma* phase was found to be stabilized by increasing temperature at *T*  >  100 *K* (based on Helmholtz free energy calculations that neglect thermal expansion effects).

The thermodynamic stability against phase segregation and formation of secondary phases of the chalcohalide compounds was assessed by means of convex‐hull DFT calculations (see Figure S4, Supporting Information). Materials presenting formation energies below the convex‐hull surface are expected to be thermodynamically stable, while the materials presenting formation energies above the convex‐hull surface (typically by ≥0.1 eV per formula unit) are expected to decompose into other compounds. Since in the experimental synthesis of chalcohalides the pure elements Bi, Sb, S, Se, I, and Br, and binary compounds M_2_Ch_3_ (M = Bi, Sb; Ch = S, Se) and MX_3_ (M = Bi, Sb; X = I, Br) are employed as the precursors, the DFT convex‐hull surfaces were built according to the formation energies of both ternary and binary compounds. For the pnictogen chalcohalides lying below the convex‐hull surface, it may be confidently assumed that they are thermodynamically stable at temperatures near ambient – recalling that DFT calculations are performed at 0 K, hence disregarding all entropy effects (although these effects typically tend to stabilize solid solutions by increasing temperature). Interestingly, the analyzed parent compounds exhibit formation energies below the convex‐hull (see Table S3, Supporting Information), indicating that they should be regarded as being thermodynamically very stable. See Figure S1c (Supporting Information) for the detailed convex‐hull surface of SbSeI.

Thin film solar cell absorbers typically exhibit narrow (i.e., ≈1 eV) direct bandgaps, along with high optical absorption coefficients within the visible range. The electronic band structure and optical characteristics of the eight chalcohalide compounds have been calculated using our DFT approach. Importantly, quantum relativistic SOC effects were found to significantly impact the conduction band of these materials, hence they have been considered for the DFT optoelectronic calculations. Also, the range‐separated hybrid functional HSE06 was used to minimize the consequences of electronic self‐interaction errors.

**Figure 4:**
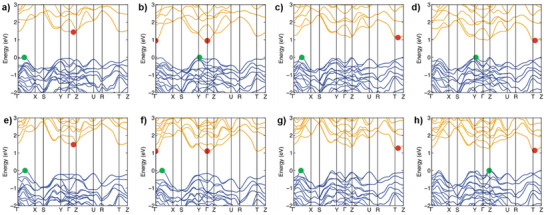
Energy‐momentum bands structures along high‐symmetry k‐paths in the Brillouin zone, computed at PBEsol+SOC level of theory. a) BiSBr, b) BiSeBr, c) BiSI, d) BiSeI, e) SbSBr, f) SbSeBr, g) SbSI, h) SbSeI.

In **Figure** **4**, conduction and valence bands are shown in yellow and blue, respectively, and the contribution of each type of orbital are presented in the corresponding density of states diagram (the Fermi energy level is set to 0 eV) – green and red dots represent the top of the valence band and the bottom of the conduction band, respectively. Significantly, the eight compounds predominantly exhibit indirect bandgaps (i.e., the VBM and CBM are not located in the same point of the reciprocal space). However, the minimum direct bandgaps are very close in size to indirect ones, presenting small energy differences of <0.2 eV between them. This small separation results in quasi‐direct bandgap behavior, which is known to enhance radiative recombination rates by reducing momentum mismatch constraints. As a consequence, stronger photoluminescence (PL) signals can be expected, even in materials that are not strictly direct bandgap semiconductors.^[^ ^55^ ^]^ Overall, the bandgaps range from 1 to 2 eV, with BiSeI and SbSBr as the compounds exhibiting the smallest and largest values respectively (1.25 and 1.93 eV). Possessing bandgaps well into the visible range, with values suitable both for single and multi‐junction solar cells, chalcohalide materials appear indeed very well suited for PV. As shown in the electronic density of states plots, p‐halogen and p‐chalcogen orbitals govern the maximum of the valence band, while p‐pnictogen orbitals are most abundant in the conduction band. **Table** **2** presents the theoretical bandgaps obtained by modelling. The importance of including SOC effects is explicitly shown by comparing the DFT bandgaps obtained at different levels of theory, see Table S4 (Supporting Information).

**Table 2:** Bandgaps obtained by DFT modelling.

Material	*E*_g,min_ [eV]
BiSBr	1.84
BiSeBr	1.30
BiSI	1.49
BiSeI	1.25
SbSBr	1.93
SbSeBr	1.45
SbSI	1.70
SbSeI	1.50

**Table** **3** summarizes the effective mass (in units of electron rest mass) of electrons and holes for each Bi/Sb chalcohalide compound, estimated for high‐symmetry k‐paths, corresponding to those depicted in Figure S5 (Supporting Information). Electron masses typically lie between 0.3 and 3.3, with 0.34 the smallest effective mass (for BiSeBr along the k‐path Λ→X), and 3.25 largest one (for SbSeI along Λ→T). On the other hand, hole masses remain between 0.2 and 2.2, with SbSI exhibiting the smallest one (0.24 for the k‐path Λ→Γ), but the largest one corresponds to BiSBr (with 2.14 along Λ→Γ). Note that effective masses from bromides, in comparison to iodides, are larger for holes and smaller for electrons. However, it does not seem to exist any particular trend in terms of the k‐path involved.

**Table 3:** Electron ($m_{e}^{*}$) and hole ($m_{h}^{*}$) effective masses for each compound, computed at PBEs+SOC level of theory, from the energy‐momentum band structures presented in Figure S4 (Supporting Information).

Material	$m_{h}^{*}$ [*m*_0_]	$m_{e}^{*}$ [*m*_0_]
BiSBr	2.14 (Λ→Γ)	1.32 (Λ→Γ)
	0.64 (Λ→X)	1.18 (Λ→Z)
BiSeBr	0.64 (Λ→Γ)	0.34 (Λ→X)
	–	1.71 (Λ→Y)
	–	0.65 (Λ→Z)
BiSI	0.46 (Λ→Γ)	1.17 (Λ→T)
	0.40 (Λ→X)	1.39 (Λ→Z)
BiSeI	0.48 (Λ→Γ)	1.96 (Λ→T)
	0.31 (Λ→Y)	1.45 (Λ→Z)
SbSBr	0.72 (Λ→Γ)	0.64 (Λ→Γ)
	0.74 (Λ→X)	0.73 (Λ→Z)
SbSeBr	1.05 (Λ→Γ)	0.38 (Λ→X)
	0.50 (Λ→X)	3.00 (Λ→Y)
	–	1.77 (Λ→Z)
SbSI	0.24 (Λ→Γ)	0.81 (Λ→Z)
	0.42 (Λ→X)	1.87 (Λ→T)
SbSeI	0.26 (Λ→Γ)	1.51 (Λ→Z)
	–	3.25 (Λ→T)

**Figure 5:**
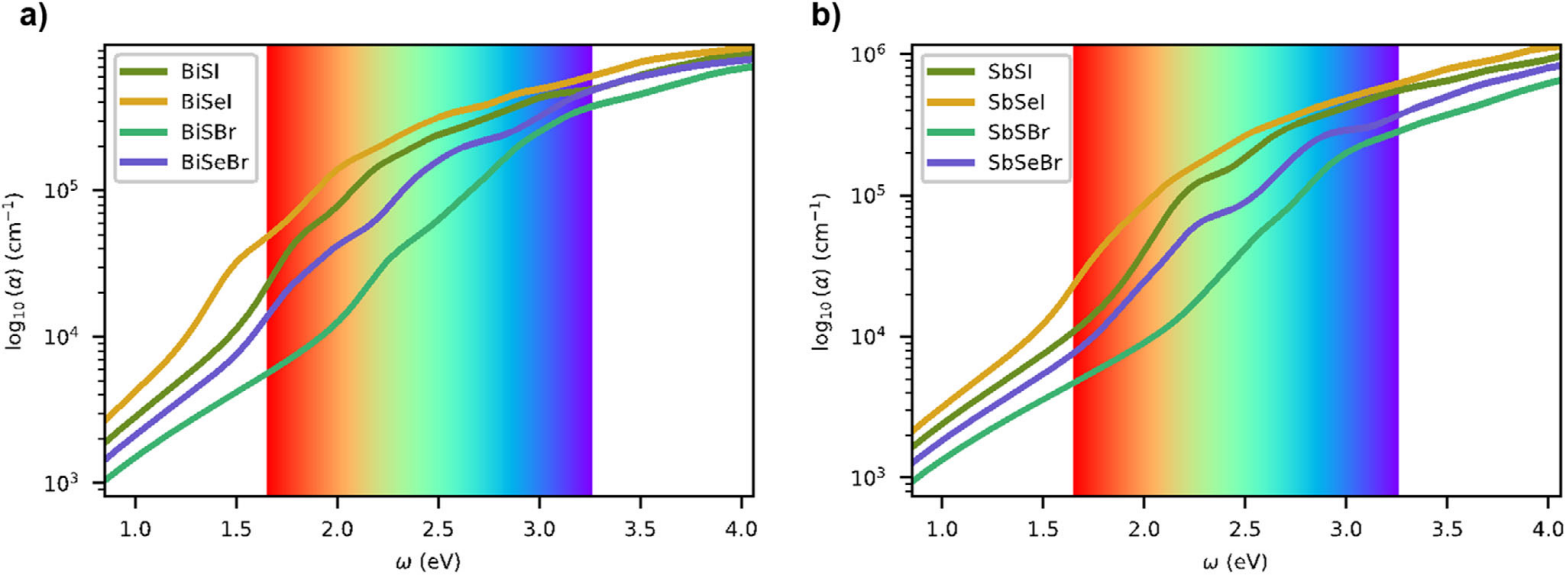
Optical absorption coefficients calculated (at 0K) for a) Bismuth and b) Antimony‐based chalcohalides. Red and blue vertical lines denote the infrared and UV limits of the visible zone, respectively. High absorption coefficients denote promising light absorbers for photovoltaic applications (of ≈10^5^ cm^−1^ just above the band gap).

In order to assess the viability of the chalcohalide family as photoabsorbers, their optical absorption coefficients (α) were calculated, see **Figure** **5**. Similarly, energy loss, extinction coefficient, reflectivity, and refractive index were determined for all the materials (see Figures S6–S9, Supporting Information). In general, high α values up to 10^5^ cm^−1^ were obtained just above the bandgap, which are highly competitive with respect to other well‐known absorbers such as Si solar cells. The highest absorption coefficient was obtained for compounds containing the following elements: Iodine < Selenium < Bismuth. In particular, BiSeI and BiSBr exhibit the smallest and largest absorption coefficients within the visible zone of 5.3 · 10^5^ cm^−1^ and 1.5 · 10^5^ cm^−1^, respectively.

**Figure 6:**
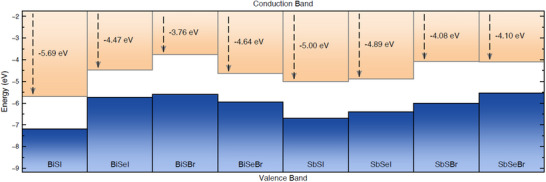
Top of the valence band (blue) and bottom of the conduction (orange) band for the chalcohalide materials family computed at the HSE06+SOC level of theory. The wide range covered by the band structures suggests promising energy applications for the chalcohalide materials family.

Finally, band positions of the eight chalcohalide compounds were computed at the HSE06+SOC level of theory, as it is shown in **Figure** **6** (see Table S5 for the VBM and CBM values). Similar trends have been found at the PBE level of theory, as shown in Figure S10 and Table S5 (Supporting Information). In general terms, the VBM lies between −7.18 and −5.55 eV while the CBM between −5.69 and −3.76 eV (as referred to the vacuum energy level). In particular, I‐based and S‐based compounds exhibit lower VBM. For instance, BiSI and SbSI present the lowest VBM (−7.18 and −6.7 eV, respectively), while BiSeBr and SbSeBr have their VBM at −5.94 and −5.55 eV, respectively. However, exceptions such as BiSBr (presenting the highest VBM at −5.6 eV) have also been found. Equivalent trends are also observed for the CBM level. It can be concluded then that the influence of the pnictogen species on the band alignments is overall limited, while the chalcogen appears the have the largest impact.

It is worth noting that several of the analyzed chalcohalide compounds emerge as potentially excellent photocatalytic materials for sunlight‐driven H_2_ production from water splitting. Besides exhibiting suitable band gaps for absorbing light within the visible range, some of the compounds also display band alignments that almost ideally straddle the hydrogen reduction H+/H_2_ (HER) and water oxidation H_2_O/O_2_ (OER) potentials – that is, −4.44 and −5.67 eV as referred to the vacuum level, respectively.

## 3 Fabrication and Fundamental Characterization

Chalcohalide thin films have been synthesized by a physical vapor deposition (PVD) method based on co‐evaporation followed by reactive annealing. Unlike previous solution‐processing techniques, the method presented here is highly reproducible and stable, and allows to obtain the complete series of (Sb,Bi)(S,Se)(Br,I) materials, including all the permutations involving metal, chalcogen, and halogen, as well as solid solutions. See a detailed description of the procedure in Methods.

**Figure 7:**
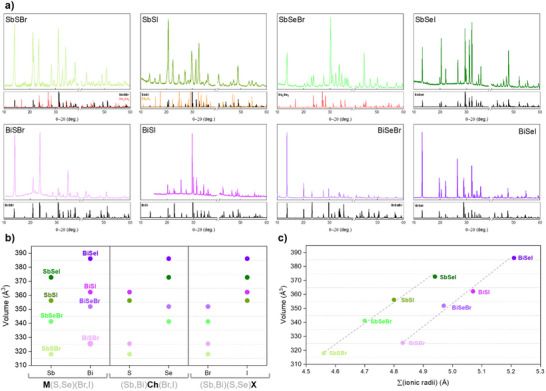
XRD analysis a) XRD patterns of the (Sb,Bi)(S,Se)(Br,I) compounds. Reference patterns included when relevant (see SI for OCD reference). b) Cell volume trends for the (Sb,Bi)(S,Se)(Br,I) compounds, illustrating how volume changes with substitution of the metal (M: Sb ↔ Bi), chalcogen (Ch: S ↔ Se), or halide (X: Br ↔ I). c) Correlation between unit cell volume and the sum of the ionic radii of the constituent elements.

**Figure 8:**
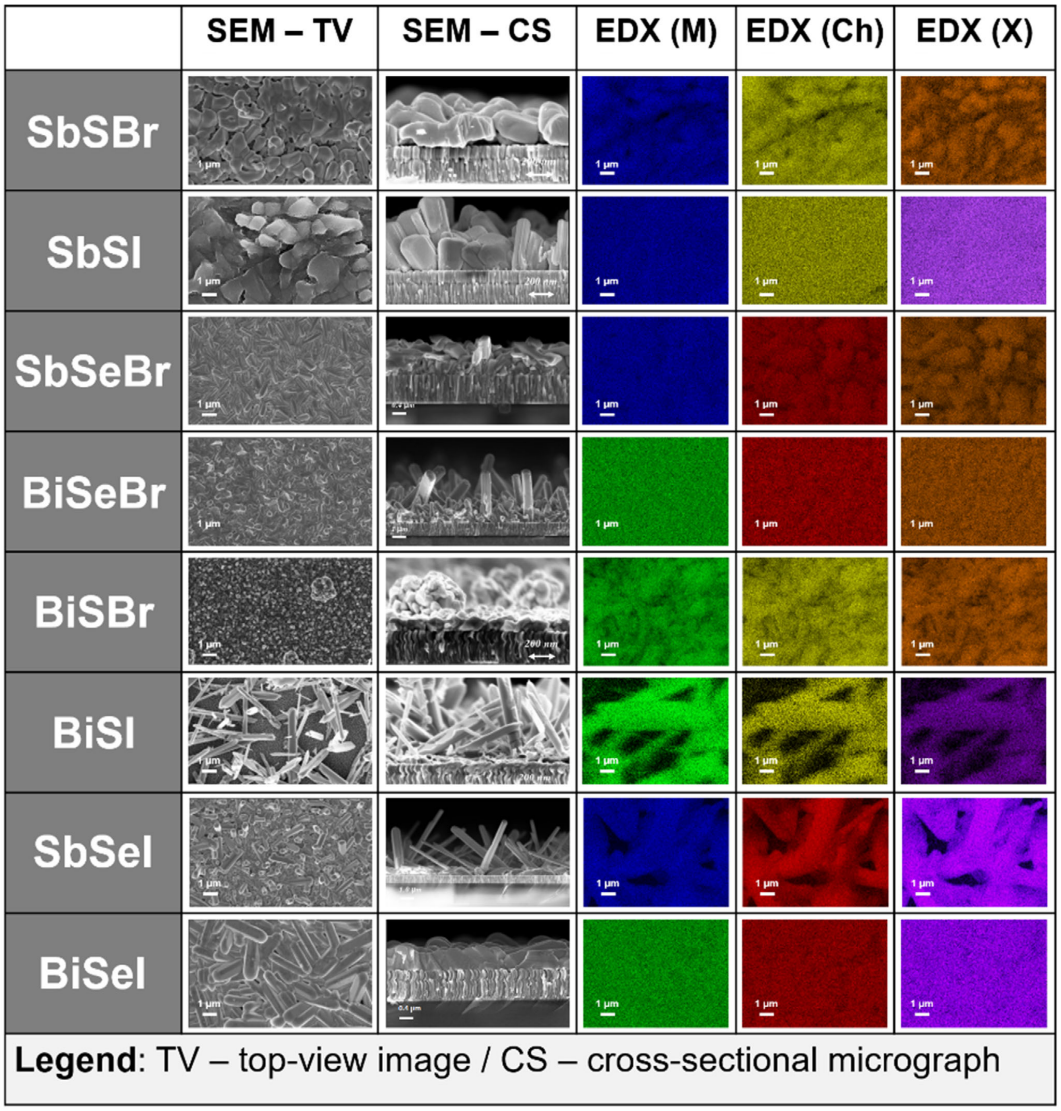
Microscopy analysis. SEM images in top‐view and cross‐section imaging modes, and SEM‐EDX elemental maps for the metal (M), chalcogen (Ch), and halogen (X).

The crystalline structure of the eight ternary MChX (M = Sb,Bi; Ch = S,Se; X = Br,I) compounds has been characterized by X‐ray diffraction (XRD), whereby diffraction patterns have been obtained showing the formation of orthorhombic *Pnma* crystalline structures, which can be indexed with the corresponding chalcohalides in the eight cases, see complete patterns in **Figure** **7**a. Also, pattern matching LeBail refinements have been performed to confirm the space group, phase identification, and to determine the structural cell parameters (see the fitted patterns in Figure S11, Supporting Information). Overall, most of the patterns present a single crystalline phase, that is, all Bragg reflections can be successfully listed to the chalcohalide orthorhombic structure with space group *Pnma*. The 39°–41° region was omitted to exclude the intense reflection from the Mo (110) plane family; this region is shown in Figure S11 (Supporting Information). Otherwise, the patterns corresponding to the SbSI and SbSeBr samples present minor signals (e.g., at 15° or 25°) that can be attributed to Sb_2_S_3_ and Sb_2_Se_3_ respectively, indicating the existence of unreacted precursor. These unreacted phases have also been confirmed by SEM imaging (see the microscopy analysis in **Figure** **8**). However, the intensity of these peaks is very low, and the dominant phase corresponds to the expected chalcohalide in all the samples.

In order to study the impact of the metal, the chalcogen, and halogen on the structural properties, cell volume and cell parameters have been determined by fitting the experimental patterns using the Le Bail refinement method, see Figure 7b (and Table S6, Supporting Information for the numeric values, and Figure S12, Supporting Information). One of the primary results here is that when a lighter element with a smaller radius is substituted for a heavier element with a larger radius (i.e., Sb to Bi, S to Se, and Br to I), the volume of the unit cell increases. However, the halides possess the biggest impact on the volume – note that all the compounds with Br have systematically smaller V than those with I, see Figure 7b. Interestingly, the trends that can be observed for the cell volume do not translate directly to the cell parameters. For instance, the halides have a bigger effect on parameters a and b, given that their discrepancy when Br is changed for I is greater than the effect that results from substituting the metal or the chalcogen. On the other hand, the chalcogenide has a significantly larger impact on parameter *c*, see Figure S12 (Supporting Information). This is a relevant result, since VdW materials such as MChX exhibit anisotropic properties originating from their “ribbon‐like” low‐dimensional structure. This unique structure emerges from quasi‐molecular ribbons linked by covalent bonds along the [00l] crystalline direction (which corresponds to the *c*‐axis), while they are held together by VdW interactions in the other directions.^[^ ^53^, ^56^ ^]^ Therefore, changing the chalcogenide, which has a bigger impact on the structural properties along the c parameter, may also have a greater effect on the properties that are conditioned by the structural anisotropy of the material, including favored growth and enhanced carrier mobility. Otherwise, the halides have a bigger impact on the crystalline directions dominated by V interactions.

Although lattice parameters typically increase when a lighter element is replaced by a heavier one, this trend does not hold for Sb/Bi substitution in the cell parameter *a*. In this case, the unit cell contracts along *a*, but expands along *b* and *c*, leading to the overall increase in volume, see Figure 7b. This behavior may result from Sb^3+^ and Bi^3+^ being larger and more polarizable cations, making them more susceptible to deformation of the electronic cloud. An alternative visual approach to illustrate the effect of substituting the elements from MChX structures on the cell parameters is to present their relative changes (whether an increase or a decrease). This is demonstrated in Figure S13 (Supporting Information). Using this representation, it becomes evident that the choice of chalcogenide exerts a pronounced influence on the dimensions of the *c‐*parameter.

Likewise, plotting the cell volume against the ionic radii reveals a clear linear trend for Sb and Bi chalcohalides, see Figure 7c. Indeed, as the ionic radii grow, the volume increases linearly for SbChX compounds, and the same trend can be observed for Bi‐based materials (BiChX). Hence, for a given metal, changes in the chalcogenide and halide compositions lead to a linear increase in cell volume as a function of the ionic radii. Note also that the order of growing radii is the same as for molecular weight. Similar increasing trends can be observed for parameters a and b; however, parameter c does not show any consistent tendency, possibly indicating a bigger impact of the chalcogen regardless of the ionic radii, see Figure S14a–c (Supporting Information). Finally, cell volume has also been plotted against the electronegativities of elements constituting each material, showing that the compounds with larger electronegativity, which correspond to the bromides (*Χ* = 2.5–2.55), have lower cell volumes (<350 Å^3^); while iodides (with *Χ* = 2.4–2.45) have a bigger volume (>350 Å^3^). It is shown that the halides have a bigger impact on parameters a and b (smaller for Br, bigger for I), supporting our previous hypothesis that VdW interactions depend strongly on the halide and metal–halide bonds, see Figure S14d–h (Supporting Information).

To sum up, the study on chalcohalides by PXRD showcases an orthorhombic *Pnma* structure for all MChX compounds. While lattice parameters generally increase when a lighter element is replaced by a heavier one, this trend is not observed for Sb/Bi substitution in parameter *a*. Here, the unit cell contracts along *a* but expands along *b* and *c*, resulting in a net increase of the cell volume. This anisotropic response highlights directional sensitivity in the structural accommodation of heavier atoms.

Additionally, the structure and composition of the eight chalcohalides have also been investigated by scanning electron microscopy (SEM) and energy‐dispersive X‐ray spectroscopy (EDX), see Figure 8. It can be noticed that the selected MChX materials tend to grow in the form of needle‐like structures, which is consistent with their characteristic quasi‐1D nature, and has been previously reported as the common morphology of chalcohalide single crystals as well.^[^ ^57^ ^]^ Particularly, the columnar morphology appears to be most notable in Se‐based chalcohalides, while sulfides present thicker, more compact grains. Nevertheless, due to this characteristic growth, some samples present a non‐uniform surface, with large areas where the substrate is clearly exposed (SbSI and BiSI), which can be detrimental for applications that require full coverage of layers (such as PV devices). Moreover, cross‐sectional images show few remnants of the unreacted precursor in the back‐contact area, especially on SbSeI, which confirms XRD observations indicating the presence of unreacted remains of the binary compounds. This suggests that the reaction likely originates from the surface of the precursor, evolving toward the substrate. Both XRD and SEM analysis show mostly homogeneous and pure‐phase chalcohalide films, with minor remnants of binary phases in certain compounds (particularly bromides), and an uneven columnar‐morphology in the cases of SbSBr and BiSI. Interestingly, SEM analysis also reveals a different morphological transformation depending on whether the precursor is Sb_2_S_3_ or Sb_2_Se_3_. For instance, it can be noted that Sb_2_S_3_ already possesses a needle‐like structure, whilst Sb_2_Se_3_ presents a thin‐film morphology.^[^ ^17^, ^56^ ^]^ Thus, after the iodination/bromination process, the sulfide‐halides appear to mostly retain the precursor's morphology, while selenides undergo a complete topological transformation. Figure 8 includes the EDX compositional maps for top‐view microscopy micrographs of the eight chalcohalides. Overall, it can be noted that the samples present an excellent compositional homogeneity, as the maps show a fully uniform distribution of the constituent elements across the entire selected surface. Based on the results presented here, the method demonstrates its potential in terms of synthesis adaptability and capability to develop high‐crystalline quality and compositionally uniform chalcohalide samples. Nonetheless, further research and optimization are required to improve the homogeneity and compactness of the layers.

**Figure 9:**
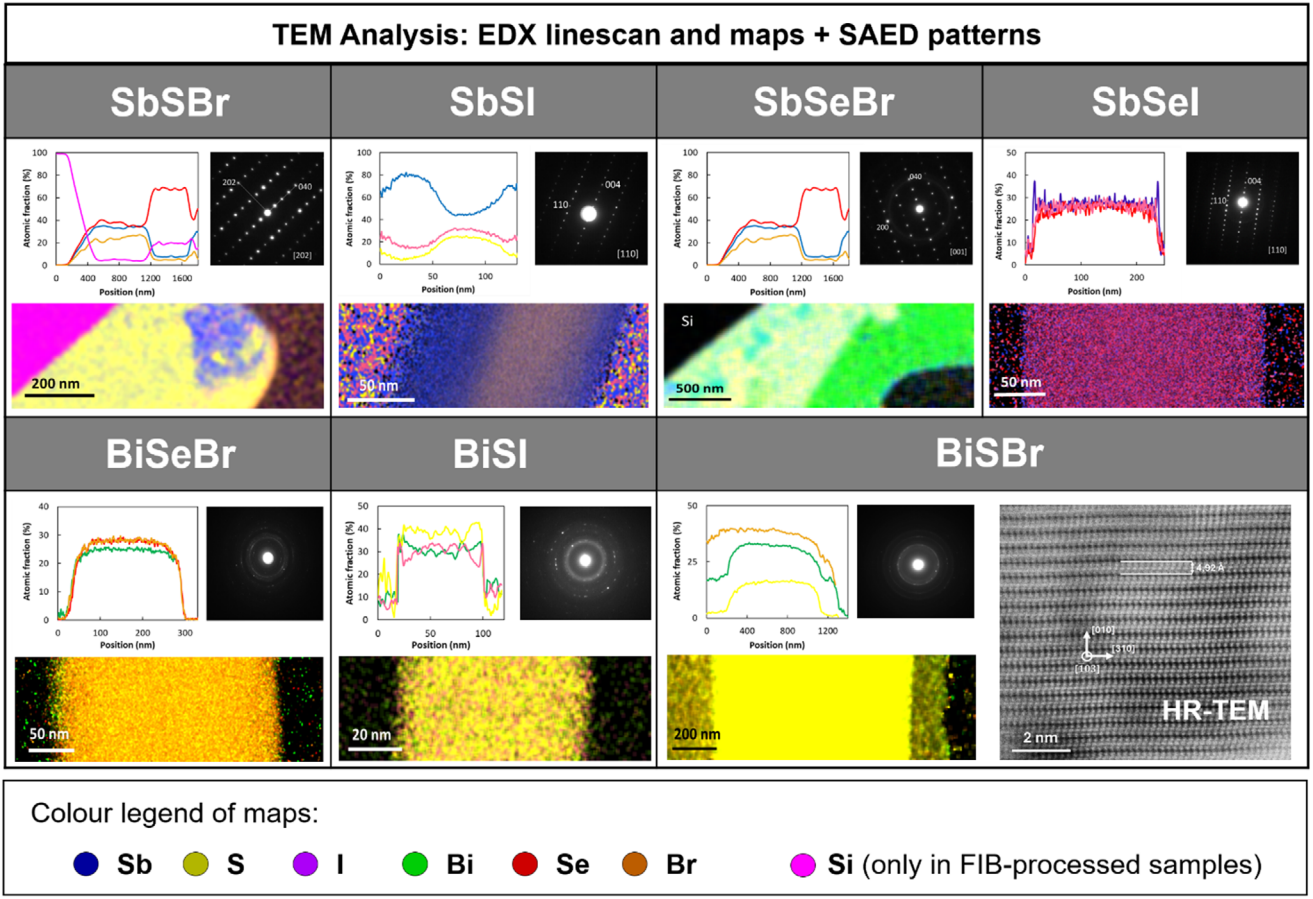
Microscopy analysis. TEM‐EDX linescans and maps, and SAED patterns of Sb‐ and Bi‐based chalcohalide thin films. Additionally, an HR‐TEM image is included for BiSBr.

Together with the previous analysis, transmission electron microscopy (TEM) cross‐sectional micrographs have been obtained, aimed at studying the structure and composition of the chalcohalide series by electron diffraction (EDP), and EDX. See Figure S15 (Supporting Information) for an overview of the sample preparation process, and **Figure** **9**a for the TEM micrographs. Interestingly, it can be observed that all Sb chalcohalides exhibit the typical EDP of a single crystal, consisting of regularly spaced bright spots, which correspond to a 2D projection of the reciprocal space crystal lattice. Analysis of the selected area pattern shows that the SbMCh samples possess an orthorhombic structure of the *Pnma* space group, validating the previous XRD analysis. On the other hand, this study demonstrates a drastically distinct growth behavior between Sb and Bi chalcohalides. While the former forms oriented single orthorhombic crystals (as shown by the EDPs), diffraction patterns of Bi compounds exhibit the characteristic ringed diffractogram of polycrystalline samples, indicating that despite a very similar needle‐shaped morphology, the mechanism involved in the formation of Sb and Bi compounds is essentially different, with the latter growing as columnar structures formed by polycrystalline domains. The mechanisms underlying these distinct crystal formation processes and their implications onto the properties of the materials remain subjects of inquiry. A thorough investigation into these phenomena is strongly recommended in the near future, as it holds significant potential to shed light to the formation mechanisms of VdW compounds and their complex structural systems.

In addition to structural analysis by EDP, TEM characterization was complemented with compositional analysis via EDX mapping. Elemental distribution maps (Figure 9) and atomic fraction line‐scans (Figure S15, Supporting Information) show that the SbSeI, BiSI, and BiSeBr samples have highly uniform compositions, with homogeneously distributed elements near the expected 33% atomic ratio each, consistent with stoichiometry. In contrast, SbSBr, SbSI, and SbSeBr exhibit compositional inhomogeneities, particularly at the edges of the columnar crystals. The cores retain stoichiometric composition, supporting the XRD and EDP findings, while their “crusts” display deviations, suggesting an outward reaction mechanism and the possible presence of an amorphous, non‐uniform outer shell – most evident in SbSeBr and SbSBr (Figure S15, Supporting Information). Remarkably, XRD patterns also reveal traces of precursor phases (Sb_2_Se_3_ and Sb_2_S_3_; Figure S11, Supporting Information), indicating incomplete conversion in these cases. Finally, HR‐TEM was performed on a high‐quality BiSBr sample, selected due to limited instrument access and quality of the layer. The HR‐TEM image reveals a highly ordered, ribbon‐like crystalline structure with periodic, well‐defined quasi‐molecular motifs in a uniform, undistorted lattice, confirming the material's intrinsically anisotropic, chain‐like microstructure.

Overall, this study demonstrates the successful synthesis of the full series of Sb and Bi‐based chalcohalides. Nonetheless, in the case of Sb bromides, the process requires optimization to minimize the presence of unreacted precursors and amorphous phases. Furthermore, it has been shown that Sb‐based compounds are composed of highly oriented single crystals, while Bi‐based compounds consist of polycrystalline structures.

**Figure 10:**
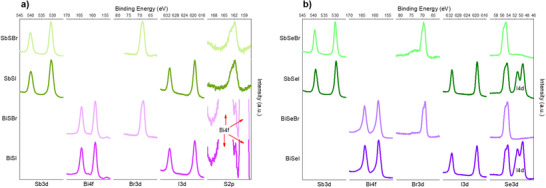
XPS analysis. Selected ranges of the spectra of chalcohalide samples. a) Sulfides, b) Selenides.

X‐ray photoelectron spectroscopy (XPS) has also been used to confirm the compositional homogeneity of the samples. See Figure S16 (Supporting Information) for the complete XPS spectra. **Figure** **10** shows selected ranges of binding energies of the XPS spectra, including the signals related to all the constituent elements – Figure 10a for sulfide films, and Figure 10b for the selenides. Importantly, signals corresponding to the elements pertaining to each material have been detected, confirming the expected composition. For example, in the case of SbSeI, doublets for the Sb3d, I3d, I4d, and Se3d orbitals have been identified, Figure 10b. In some instances, the difference in binding energy of the doublet is very small, resulting in such peaks appearing superimposed; for example, Se3d overlapped peaks appear as a single signal with a shoulder in the higher energy region. Otherwise, no signals unrelated to the expected elements are detected, and no shifts in their positions are observed, which would suggest the absence of phases with different oxidation states or chemical environments, hence proving the remarkable chemical homogeneity of all the samples.

Regarding the spectra of sulfides (Figure 10a), we note that the signals corresponding to sulfur orbital S2p are completely overlapped by the Bi4f peak, which is much more intense. However, in the magnified spectra (range 168–159 eV), it can be seen that to the left of the highest energy peak of the Bi4f doublet, there is a lower intensity peak that perfectly coincides with the low energy signal of the S2p doublet, confirming the presence of sulfur. Additionally, we note that the Bi signals show a slight asymmetry.

**Figure 11:**
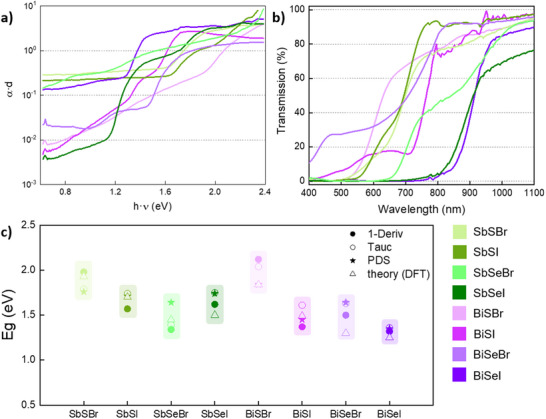
Optical spectroscopy analysis. a) Absorption spectrum (PDS) and b) Transmission spectrum (UV–vis spectroscopy) of the eight MChX compounds. c) Comparative plot of the bandgap of the eight MChX compounds: values calculated by PDS, UV–vis transmission spectroscopy (Tauc and first‐derivative methods) and first‐principle DFT calculations.

The optical properties of the eight MChX chalcohalide compounds, including bandgap and absorption coefficient, have been studied by transmittance UV–vis spectroscopy and Photothermal Deflection Spectroscopy (PDS). In order to determine the absorption coefficient (α) from the absorptance spectra measured by PDS, an exponential rule is applied according to which α is directly proportional to the product of 1/*d* (where *d* is the thickness) and the natural logarithm of absorbance.^[^ ^56^ ^]^ However, due to the very rough morphology of the chalcohalide samples, it has not been possible to determine a uniform thickness *d*. Alternatively, the absorption spectrum has been represented as the product *α·d*, see **Figure** **11**a. Importantly, PDS allows highly precise optical measurements in the sub‐gap range, offering valuable metrics to evaluate the quality of the material, such as the presence of sub‐bandgap transitions; typically attributed to defect states. Interestingly, Figure 11a shows that for sulfides (MSX), Bi compounds present lower sub‐gap absorption, indicating a minor impact of optical transitions in the sub‐gap zone (e.g., defects). On the other hand, in the case of MSeX, the lowest absorption overall belongs to SbSeI, suggesting that the chalcogenide has a tangible and differentiated effect on the defect properties of this family of materials. Also, it can be noted that iodides present better‐defined absorption fronts (lower slope indicating lower Urbach energy) than bromides, likewise suggesting that halides beget a significant impact on Urbach tails. In particular, bromides lead to higher Urbach energies, which in other cases have been proven to correlate directly with a negative effect on carrier mobility and lifetime.^[^ ^58^ ^]^ Otherwise, the multistep front that can be noted in some spectra (e.g., SbSeI) indicates that these samples include secondary phases resulting in multiple bandgaps. For instance, in the case of SbSeI, the absorption drop corresponds to ≈1.2 eV, which is compatible with Sb_2_Se_3_’s bandgap, confirming that there are traces of precursor remaining in the sample. Finally, the bandgap of the eight chalcohalides has been calculated by applying a combined Urbach–Tauc model^[^ ^59^ ^]^ revealing that lighter elements lead to higher bandgaps: *E*_g,S_ > *E*_g,Se_/*E*_g,Br_ > *E*_g,I_/*E*_g,Sb _> *E*_g,Bi_, see Figure 11c.

Additionally, UV–vis transmission spectroscopy has been used to acquire the transmission spectra of the MChX compounds, see Figure 10b. The absorption front is well defined, and it is possible to note an increasing trend of *E*_g_ (absorption front shifts to smaller wavelengths) as the compounds become lighter. Optical bandgaps have been calculated applying the Tauc and first‐derivative methods, see Figure 11c. Furthermore, the calculated bandgap by DFT (see Table 2) has been included in Figure 11 as well, for the sake of comparison. Importantly, it is shown that regardless the method, the relative trends discussed are consistent, although some techniques result in larger variations between different compounds (first‐derivative). However, in most cases, the bandgap variations obtained by different methods do not exceed 0.2–0.3 eV. The consistency shown between theoretical values and measurements validates the methodology used to compute the optical properties by DFT. Otherwise, the bandgap does not undergo a major difference when changing some elements with respect to the others, but rather there is a combined effect that leads to the observed variations within a range between 1.2 and 2.2 eV. These results are significant, since they demonstrate that MChX compounds cover a broad bandgap range depending on the composition, opening the door to developing applications for specific optical properties. For instance, wide bandgap chalcohalides such as SbSI or SbSeI (*E*_g_ ≈ 1.6–1.8 eV) are optically compatible for top cell tandem PV devices, while lower bandgap compounds (1.2–1.6 eV) possess optimum properties for single‐junction device solar cells and photocatalytic applications. Furthermore, bandgap tuning could be achieved by developing solid solutions (Sb,Bi)(S,Se)(Br,I), resulting in a continuous range which can be adjusted by controlling the relative amounts of each element.

**Figure 12:**
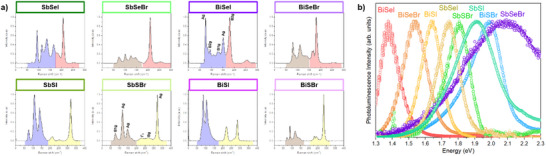
Raman and PL spectroscopy analysis. a) Raman spectra and b) PL spectra of the eight MChX compounds.

Raman spectroscopy analysis has been performed for the full set of chalcohalides materials, see **Figure** **12**a. Significantly, this is the first time that the set of eight ternary compounds has been studied consistently together by Raman spectroscopy. In particular, this is the first report including the complete Raman spectra of SbSeBr and BiSeBr, to the best of our knowledge. See Table S7 for a list of the main peaks and fitted FWHM acquired from the Figure 12a spectra. Importantly, we note that all the spectra can be divided into 2 ranges:

From 70 to 117 cm^−1^: Sb/Bi‐X_1_ (X_1_ = Br, I). Corresponds to the halogen–pnictogen interactions, highlighted in blue (M‐I) and brown (M‐Br).

From 118 to 332 cm^−1^: Sb/Bi‐X_2_ and X_2_‐X_2_ (X_2 _= S, Se). Corresponds to chalcogen‐pnictogen and Ch–Ch interactions, highlighted in red (Se) and yellow (S).

The characteristic Raman modes for each spectrum have been confirmed based on literature references, see Table S7.^[^ ^60^, ^61^, ^62^, ^63^, ^64^, ^65^, ^66^, ^67^, ^68^, ^69^, ^70^, ^71^, ^72^ ^]^ Most spectra display common features attributed to halogen–pnictogen and chalcogen–pnictogen interactions. Several compounds (e.g., SbSBr, SbSI, BiSI) show three prominent peaks under 175 cm^−1^, typically associated with A_g_ and B1_g_ symmetries, as previously reported for SbSBr and BiSeI.^[^ ^65^, ^73^ ^]^ Sb–Se interactions produce a peak above 200 cm⁻¹, while the corresponding Bi–Se peak appears slightly below this value. In contrast, Sb–S and Bi–S modes occur at higher shifts, slightly above and below 300 cm⁻¹, respectively. Comparing samples with the same chalcogen but different pnictogens, a minor redshift is observed in the main M–Ch peaks upon replacing Sb with Bi; for example, from 321 to 318 cm^−1^ in SbSBr versus SbSI, and from 283 to 284 cm^−1^ in the Bi analogs. This shift, also noted by Groom et al.^[^ ^64^ ^]^ reflects the effect of pnictogen substitution. A similar trend is seen in Se‐based compounds: the Raman shift changes from 207 to 219 cm^−1^ for SbSeI and SbSeBr, and from 181 to 180 cm^−1^ for their Bi counterparts. Reference Raman modes for SbSBr and BiSeI are shown in Figure 12, as taken from the literature. However, further work is needed to unambiguously assign Raman modes for the other compounds.

Finally, PL spectra were obtained at room temperature for the series of eight chalcohalides. Again, this is the first time that certain materials, like SbSeBr, have been characterized by PL. The spectra present overall sharp and intense peaks, with the exception of SbSeBr and SbSI, which exhibit broader bands. The fact that these compounds presented the largest sub‐gap absorption and Urbach energy (see PDS spectra in Figure 12a), together with the broad PL signals, indicates that they possess a high density of sub‐bandgap states, which can result from bandgap fluctuations, band tailing, and potentially detrimental trap and defect states. Otherwise, the maximum of the PL spectrum (see values in Table S8, Supporting Information) presents the following decreasing order: *E*_SbSeBr_ > *E*_BiSBr _> *E*_SbSI _> *E*_SbSBr_ > *E*_SbSeI _> *E*_BiSI _> *E*_BiSeBr _> *E*_BiSeI_, which correlates generally well with the bandgaps optically measured by PDS: *E*_BiSBr _> *E*_SbSBr‐ _> *E*_SbSeI _> *E*_SbSI _> *E*_BiSeBr _> *E*_SbSeBr_ > *E*_BiSI _> *E*_BiSeI_.

The optical properties of the eight MChX compounds have been explored, revealing how the elemental composition influences bandgap and absorption. Variations in sulfides and selenides highlight their differing effect on sub‐bandgap transitions. Otherwise, halides affect the bandgap tail and Urbach energy. Also, these results validate the calculation methods, showcasing consistent results and a wide bandgap range (1.2–2.2 eV). Likewise, exploring solid solutions offers potential for customizable bandgap tuning.

**Figure 13:**
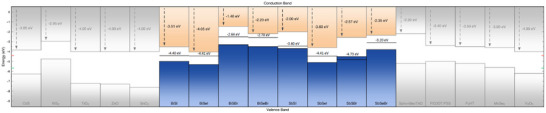
Top of the valence band (blue) and bottom of the conduction band (orange) for the chalcohalide materials family obtained by UPS (VB), with the CB determined by considering the optical bandgap. The Fermi level is shown as a horizontal line, also indicating the work function with respect to vacuum. The figure also shows examples of various ETL (left) and HTL (right) layers – see Supporting Information for the list of references used for these values. Red and green lines on each side of the plot indicate the OER and HER potentials, respectively.

UPS has been used to determine the VBM (in Figure S17, Supporting Information the complete VB measurements). **Figure** **13** shows the band alignments of the eight chalcohalides, as measured by UPS from the Mo/MChX layers. UPS is a powerful spectroscopic technique that allows measuring energies of VB states of metals and semiconductors with respect to the vacuum level; however, it does not provide the CBM positions. In order to present the whole band structure, CBM has been determined from the UPS–VBM measurements together with optical bandgaps by PDS. Thus, it is important to recognize that the VB and CB measurements have been obtained using different methods based on distinct physical principles. In particular, UPS relies on measuring the energy of the photoelectrons emitted by a material, whereas PDS measures its absorbance spectrum. Thus, the final state of the material in terms of electronic distribution in both cases is different, as the VB is obtained after an ionization process. Considering that variations in the final electronic distribution do not imply significant changes in the relative position of the bands with respect to the vacuum, we consider that the band diagram in Figure 13 provides an acceptable estimation for comparing relative alignment between materials.

**Figure 14:**
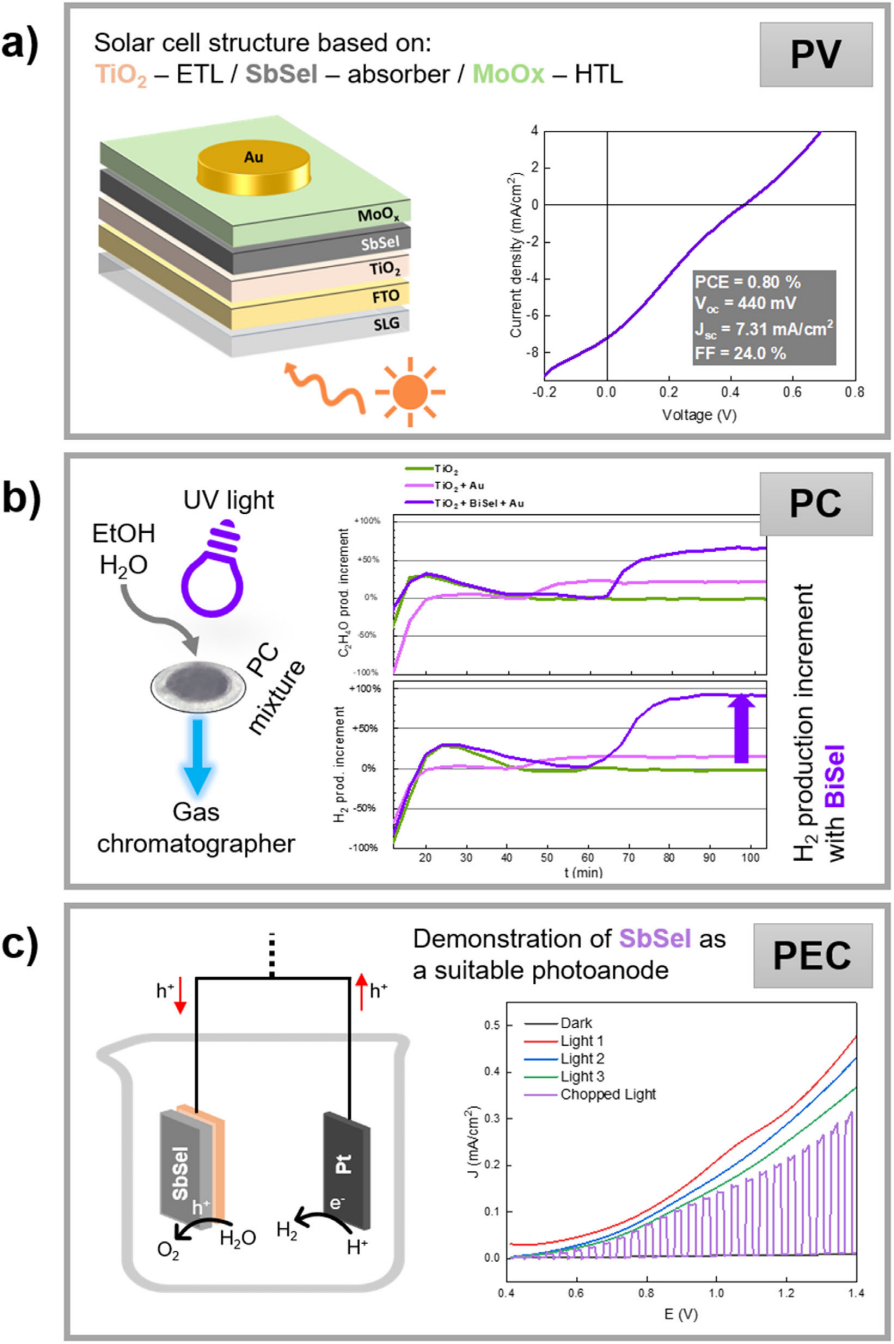
Overview of MChX‐based applications across three domains: a)PV. Device architecture and performance of an FTO/TiO_2_/SbSeI/MoO_x_/Au solar cell, with corresponding JV curve and optoelectronic parameters. b)PC. Ethylene glycol (C_2_H_4_O) and hydrogen (H_2_) evolution enhancement using BiSeI+TiO_2_+Au photocatalyst powder under UV+Vis light, shown via product increment graphs. Also includes a schematic of the experimental setup. c) PEC. JV curves of a SbSeI/TiO_2_/SLG photoanode under dark, continuous, and chopped simulated sunlight, demonstrating enhanced photocurrent under illumination. Also includes a schematic of the PEC setup.

Overall, it is observed that the relative trends between the band positions calculated by DFT and those measured experimentally are indeed very similar, see the calculated band structure in Figure 6. For example, in both instances, the material with the bands closest to the vacuum level is BiSeBr, whereas the increasing VB trend for SbSeI < SbSBr < SbSeBr is also detected in both the simulation and measurements. Otherwise, it is worth noting that the experimentally measured values generally show bands closer to the vacuum level, with a consistent shift toward smaller values (in absolute terms). This variation may be due to several reasons: first, DFT calculations were performed under 0 K conditions, whereas measurements take place at room temperature. Second, previous experiments suggest that chalcohalide materials synthesized by the process presented in this work exhibit n‐type conductivity (see I. Caño et al.),^[^ ^17^ ^]^ which implies the potential existence of donor‐type structural defects (Se/S vacancies) that are not considered when simulating the material, as these assume a perfect crystalline structure. These considerations might explain the shift observed between Figures 6 and 13, although we wish to highlight that relative differences suggest a good match between calculations and experiments.

Additionally, UPS measurements have been used to determine the work function (WF) of the eight materials. Since the measurements are referenced to the vacuum level, the WF allowed the calculation of the Fermi level energy, as shown in Figure 13. Regarding this parameter, it is observed that most materials have a Fermi level very close to the center of the bandgap, indicating that they are essentially intrinsic. However, BiSeI and SbSI appear to be strongly n‐type and p‐type doped, respectively, and the Fermi energy located within the VB for SbSBr suggests that this material exhibits metallic behavior. Otherwise, it is noteworthy that SbSeI shows a slight n‐type doping, confirming previous evidence obtained through current–voltage measurements on SbSeI crystals with metal electrodes of different WF.^[^ ^17^ ^]^ From these results, it can be inferred that S‐based compounds are predominantly p‐type, whereas Se‐based compounds are either n‐type or intrinsic. This difference may arise from the extent of chalcogen incorporation: in Se‐based materials, chalcogen may not integrate as effectively, leading to a slight excess of pnictogen. Conversely, S‐based compounds exhibit a chalcogen excess. According to DFT studies on binary chalcogenides, the dominant defects under metal‐rich conditions (e.g., Sb_Se_ and V_Se_) act as n‐type dopants, while under chalcogen‐rich conditions, p‐type defects (e.g., Se_Sb_) prevail.^[^ ^74^ ^]^ Based on this interpretation, fine‐tuning the pnictogen‐to‐chalcogen ratio is essential for achieving effective control over semiconductor doping.

Finally, Figure 13 also provides useful insights to explore the possibilities of various materials for optoelectronic applications. For example, BiSBr, with VB and CB positions lying very close to the vacuum level, is not particularly attractive for photovoltaic applications, given that for most ETLs (electron transport layer), a cliff‐type interface would form, resulting in losses due to recombination; see band position of standard ETLs in Figure 13‐left. On the other hand, BiSeI and SbSeI (VBM at −5.4 and −5.3 eV respectively, and CBM −4.1 and −3.6 eV) exhibit an optimal band alignment. Comparing their band positions with those of various semiconductors commonly used as ETL and HTL (hole transport layer), we notice that BiSeI and SbSeI present adequate alignment with several ETLs such as CdS, TiO_2_, and ZnO. Otherwise, Spiro‐MeoTAD is an optimal HTL, see Figure 13‐right. Hence, BiSeI and SbSeI appear to be the most promising chalcohalides for PV applications, showcasing optimal band alignment with CdS and oxide ETLs, and Spiro‐MeoTAD presenting good positioning for HTL.

Based on the different band alignments, all these materials also exhibit the potential for green hydrogen production through water splitting. The thermodynamic energy required to split water into its elemental constituents is 1.23 V. For effective PC, the material's CBM must lie at a more cathodic potential than the water reduction potential (−4.44 eV), whilst the VBM must be positioned at a more anodic potential than water's oxidation potential (−5.67 eV). This alignment ensures that photoexcited charge carriers possess sufficient potential to drive the splitting of water molecules into H₂ and O₂. Based on these criteria, materials such as BiSeI and SbSBr have been identified as promising photocatalysts. See Section 4 for further insight on the potential of BiSeI for green hydrogen generation applications. Moreover, water‐splitting efficiency can be enhanced by spatially separating the reduction and oxidation reactions across two different semiconductors (PEC). This approach divides the photovoltage required for water splitting between both materials, hence mitigating surface recombination losses and enabling higher current densities. In this configuration, the semiconductor employed as a photocathode only requires its CBM to be more cathodic than the water reduction potential, whilst the photoanode requires its VBM to be more anodic than water's oxidation potential. Considering these additional design principles, materials such as SbSeBr, BiSBr, BiSeI, and SbSBr could be promising candidates as photocathodes, whereas BiSeBr, SbSeI, BiSI, SbSI, BiSeI, and SbSBr are suitable for photoanodes.

## 4 Functional Optoelectronic Applications

Recent progress in Sb and Bi chalcohalides has demonstrated their remarkable versatility for optoelectronic applications such as PV, photodetectors, and PEC. Materials like SbSI (PV efficiencies >3%)^[^ ^75^ ^]^ and BiSI (photodetector responsivity of 62.1 AW^−1^ at 10 V),^[^ ^27^ ^]^ demonstrate their promise for green energy technologies. However, research has largely focused on a narrow subset of compositions (primarily SbSI), with many potential applications still unexplored. In this context, our work introduces a robust, physical methodology enabling the fabrication of a wide array of Sb and Bi chalcohalides, extending their application to PV, PC, and PEC. This includes the first demonstration of PC activity using BiSeI, as well as novel PV and PEC devices based on SbSeBr and SbSeI.

With optimal bandgaps (1.2–2 eV) and high absorption coefficients, Sb and Bi chalcohalides are naturally suited for solar energy conversion. Even with limited literature, PV prototypes have shown encouraging performance, including reported PCEs of 4.1% for SbSeI,^[^ ^18^ ^]^ 3.05% for SbSI,^[^ ^20^ ^]^ and 1.3% for BiSI.^[^ ^24^ ^]^ Notably, these devices were produced via chemical routes, such as solution deposition. In contrast, our physical synthesis methodology provides a more adaptable and scalable approach, yielding working PV devices with SbSeI and SbSeBr, with initial efficiencies between 0.3–0.6%.^[^ ^17^ ^]^ Significantly, this includes the first SbSeBr‐based solar cell reported in the literature. These devices exhibit high open‐circuit voltages (≈600 mV), suggesting strong potential for PV applications. However, substrate‐based architectures (glass/Mo/MChX/CdS/ITO)^[^ ^56^ ^]^ suffer from poor current extraction (*J*_SC_) due to the columnar morphology, which causes non‐optimal contact with the CdS layer and results in low shunt resistance. Interestingly, SbSeBr's more compact microstructure yielded better performance, underlining the impact of morphology on device efficiency. Also, electronic characterization revealed n‐type conductivity in SbSeI, rendering it less compatible with the n‐type CdS‐based architectures.

To address this mismatch, here a superstrate configuration has been developed based on the following architecture: glass/FTO/TiO_2_/SbSeI/MoOx/Au, utilizing F‐doped SnO_2_ (FTO) as the front contact, TiO_2_ as the ETL, and MoOx as the HTL (see Methods for the complete procedure of device preparation). Devices fabricated in this structure showed a *V*_OC_ above 440 mV and a PCE of 0.8%, the highest achieved to date using this synthesis method, see **Figure** **14**a. Despite this progress, low *J*_SC_ and FF (<25%) reveal limitations in carrier extraction and interface quality. These results emphasize the need for continued material optimization, specifically improving carrier concentration, reducing defect densities, and developing enhanced ETL/HTL layers, better aligned with chalcohalide electronic structures.

PC is a promising technology aimed at developing environmentally‐friendly energy applications, which involves harnessing light to drive chemical reactions. Remarkably, BiSeI stands out as a promising photocatalyst due to anisotropic columnar structure, covalent ribbon bonding, and a bandgap suitable for visible light activation, properties that support both solar and photocatalytic performance. The band gap of BiSeI (at ≈1.25 eV), determines a big step forward compared to TiO_2_, the most studied compound for PC so far, but inherently limited by a ≈3 eV bandgap.

To assess its photocatalytic activity, BiSeI was incorporated with TiO_2_ and Au nanoparticles (NPs) for ethanol dehydrogenation. Three samples: TiO_2_, TiO_2_ + Au NPs, and TiO_2_ + Au NPs + BiSeI powder, were tested under UV and UV–vis illumination (see Methods for a complete description of the procedure to prepare the samples and perform measurements). As shown in Figure 14b, only the mixture containing BiSeI (purple line) exhibited a dramatic increase in activity, with H_2_ production rising by 91% and C_2_H_4_O by 68% under UV–vis light compared to UV alone. No CO_2_ or CO were detected, confirming selective ethanol‐to‐hydrogen conversion. This remarkable enhancement is attributed to efficient charge separation and the low bandgap of BiSeI, leading to improved photocatalytic activity. These results establish the first successful demonstration of BiSeI‐based PC for H_2_ production.

Finally, SbSeI also shows promise as a photoanode for water splitting due to its ≈1.7 eV bandgap, n‐type conductivity, and nano‐columnar morphology. These properties, along with its favorable band alignment (see Figure 12), suggest potential as a tandem photoanode material and a possible alternative to the commonly used BiVO_4_. PEC performance tests were performed using glass/FTO/TiO_2_/SbSeI devices in a neutral electrolyte solution, using Pt as the counter‐electrode (see Methods for detailed description of the experimental setup). The measurements produced a photocurrent density of 0.35 mA cm^−2^ at 1.2 V versus RHE and an onset potential of 0.4 V, even without co‐catalyst modification, see Figure 14c. This marks the first proof‐of‐concept for SbSeI as an OER‐active photoanode.

## 5 Conclusion

This work has introduced a systematic and versatile methodology to fabricate the full suite of (Sb,Bi)(S,Se)(Br,I) chalcohalides, enabling their synthesis with reproducible properties and a high compositional uniformity. The study demonstrates that all these compounds possess an orthorhombic phase with space group *Pnma*, growing as needle‐shaped crystals – indicative of ribbon‐like structure –, with the chalcogenide having the biggest impact on the direction of growth. Microscopy analysis reveals high crystalline quality and compositional homogeneity. Significantly, the extensive characterization study has shown that several properties depend directly on composition, allowing them to be classified according to the metal or chalcogen. Specifically, distinct growth mechanisms have been discovered between Sb and Bi‐based compounds, leading to single‐crystal and polycrystalline morphologies, respectively. Additionally, UPS measurements have shown that S‐based compounds are predominantly p‐type, whilst Se‐based compounds are either n‐type or intrinsic, indicating that the dominant formation of different defects is chalcogen‐dependent. Finally, optical characterization has provided the bandgaps of all materials, spanning a broad range from 1.2 to 2.2 eV, and a complete Raman symmetry analysis of all chalcohalides has been performed for the first time. Importantly, characterization work has been corroborated by material simulations based on DFT, confirming the stability of the orthorhombic phase and revealing a strong agreement between the optical properties obtained from calculations and experiments. Notably, relative trends and variations are consistent across both methods.

Second, the materials have been successfully integrated into functional devices for PV, PC, and PEC, marking a significant step forward in their implementation for sustainable energy solutions. The substantial increase in photocatalytic activity and demonstrated photoelectrocatalytic response underscore their suitability for green hydrogen production and other energy‐related applications. By showcasing the unique quasi‐1D VdW structure, defect‐tolerant electronic properties, and tunable optoelectronic characteristics, this work highlights the chalcohalide family's relevance for advanced energy and optoelectronic applications. Indeed, these findings pave the way for the development of scalable, eco‐friendly materials tailored for next‐generation technologies, from PV to green hydrogen generation.

## 6 Experimental Section

### First‐Principles Calculations

*Ab initio* calculations based on DFT were performed to analyze the physical and chemical properties of bulk MChX chalcohalide materials. The calculations were performed with the VASP package following the generalized gradient approximation to the exchange‐correlation energy (J.P. Purdew, K. Burke, M. Ernzerhof). The projector augmented‐wave method was used to represent the ionic cores, and for each element, the maximum possible number of valence electronic states was considered. Wave functions were represented in a plane‐wave basis typically truncated at 850 eV. By using these parameters and dense **k**‐point grids for Brillouin zone integration, the resulting zero‐temperature energies were converged to within 1 meV per formula unit. In the geometry relaxations, a tolerance of 0.005 eV Å^−1^ was imposed in the atomic forces. For each compound, a primitive cell with 12 atoms was selected the Brillouin zone of which was sampled with an 11  ×  5  ×  4 Γ‐centered mesh (in reciprocal space).

As the baseline, the PBEsol functional (a revised version for solids from the Perdew–Burke–Ernzerhof functional), was used for all calculations. Given the importance of VdW forces in these materials, long‐range interactions were not negligible; therefore, geometry optimizations included the VdW corrections. Quantum relativistic effects on the electronic bands were also taken into consideration (i.e., spin‐orbit coupling corrections), for optoelectronic calculations along with hybrid functionals (HSE06+SOC). Phonon calculations were performed with the small‐displacement method and the PHONOPY software. Large supercells (324 atoms) constructed as 3  ×  3  ×  3 unit cell replications were employed along with a **k**‐point grid of 4  ×  2  ×  2 for sampling of the Brillouin zone.

For an accurate estimation of the band alignments, the electrostatic potential of the valence band maximum (VBM) and vacuum level of the bulk materials were calculated. The absolute energy level of the VBM relative to the vacuum was defined as the difference between the VB energy and the vacuum level. Conduction Band Minimum (CBM) energy was determined by adding the bandgap (*E*_g_) to the previously calculated VBM energy (at the HSE06 level):

(1) $$\mathrm{CBM}\;=\;\mathrm{VBM}\;+E_{g}$$

A slab‐vacuum model of 20 Å thickness and 20 Å vacuum along the *c* direction was employed for the VBM calculations. To refer energies to the vacuum level, two different macroscopic average potential calculations were required for each material: one for the bulk and the other for the material in contact with vacuum (i.e., slab calculation). From them, on one hand, the position of the VBM relative to the average local potential in the bulk (VBM = VBM^DFT^ − *V*_bulk_) and, on the other hand, the difference between the average local potential and the vacuum level (*V*_bulk_ − *V*_vacuum_) could be extracted. By combining these two quantities, the VBM energy relative to the vacuum level was obtained simply as follows:

(2) $$VBM=VBM^{DFT}-V_{bulk}+\Delta V_{\mathrm{vac}}$$

### Synthesis of Chalcohalide Materials

Chalcohalides thin films were synthesized by a PVD method based on co‐evaporation followed by reactive annealing. Unlike previous solution‐processing techniques, the method here presented was highly reproducible and stable, and allowed to obtain the complete series of (Sb,Bi)(S,Se)(Br,I) materials, including all the permutations involving metal, chalcogen, and halogen, as well as solid solutions. The 2‐step method here presented consisted on the following procedures:

1. Binary chalcogenide synthesis by co‐evaporation (Sb_2_Se_3_, Sb_2_S_3_, Bi_2_Se_3_, and Bi_2_S_3_).
2. The binary chalcogenide precursor is subjected to reactive annealing under high‐pressure halide atmosphere to obtain the ternary chalcohalides (MChX).

**Figure 15:**
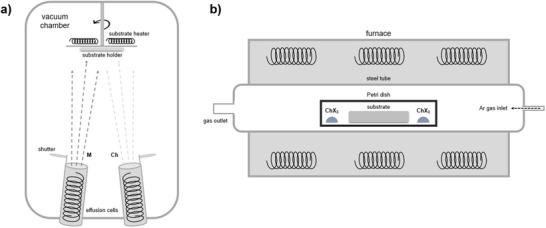
a) Scheme of the co‐evaporation process. b) Scheme of the tubular furnace for reactive annealing.

The binary chalcogenide was deposited on commercial glass/Mo substrates by co‐evaporation from elemental sources of Sb (Sigma‐Aldrich, 100 mesh, 99.5%), Bi (Sigma‐Aldrich, 100 mesh, 99%), Se (Thermo‐Scientific, 200 mesh, 99.9%), and S (Thermo‐Scientific, flakes, 99.9%). To obtain Sb chalcogenides, the metal was heated at 550 °C; alternatively, to obtain Bi compounds, the metal was heated at 680 °C. Importantly, in these experiments, the substrate was heated at 280 °C, enabling the metal and the chalcogen to react immediately upon their deposition onto the substrate, which results in the chalcogenide formation. The procedure is designated as a co‐evaporation, as it involves the simultaneous and coordinated thermal evaporation of both precursors (metal and chalcogen), and the desired chalcogenide is obtained in situ, by heating the substrate during the deposition, see **Figure** **15**a.

In the second step, the chalcogenide precursor M_2_Ch_3_ is subjected to reactive annealing in a steel tubular furnace under halogen atmosphere – using SbI_3_, SbBr_3_, BiI_3_, or BiBr_3_ as halide source. Hence, the glass/Mo/M_2_Ch_3_ samples are placed into Petri dishes which contain the corresponding halide; for example, Sb_2_Se_3_ was placed in the Petri dish along with SbI_3_ to obtain SbSeI. Subsequently, the Petri dish was introduced into the tubular furnace with inert Ar atmosphere, which was then subjected to a high temperature and high‐pressure annealing, see Figure 15b. See **Table** **4** for a complete summary of temperature, pressure, and annealing conditions used to obtain each chalcohalide. These parameters corresponded to the processing conditions determined after a series of tentative experiments, as they best aligned to the goal of achieving each compound under optimal conditions. Nevertheless, it was underscored that this study was exploratory in nature, and further comprehensive optimization for specific applications would be required in future work.

**Table 4:** Summary of the annealing conditions for the fabrication of the eight chalohalides. P_i_ indicates initial pressure (pressure at 20 °C).

Material	*T* [°C]	*P*_i_ [bar]	*t* [min]
SbSeI	450	3	15
SbSeBr	450	4	15
SbSI	300	2	15
SbSBr	325	2.5	15
BiSeI	500	4	15
BiSeBr	450	4	15
BiSI	425	2.5	15
BiSBr	400	2.5	15

Hence, these experiments were performed using a steel tubular furnace which allows annealing conditions with working pressures above 1 atm, up to 100 atm (although for the purposes of this work, pressure was limited to 20 atm). Accordingly, the novel synthesis of Sb and Bi chalcohalides was developed by varying temperature, annealing time, and pressure conditions. This method opened the door to exerting greater control over the kinetics and reactivity of the halide precursors, which were typically volatile, and also allowed to tune the morphology and purity of the final products. Moreover, this was a reproducible and facile method to synthesize bromides (such as SbSeBr), which were proven to be extremely difficult to obtain by solution‐processing routes.

### Preparation of Prototype Devices

Solar cells were prepared in superstrate architecture with the following stack structure: glass/FTO/TiO_2_/MChX/MoO_3_/Au. First, a 30 nm TiO_2_ layer was deposited onto a commercial glass/FTO substrate (Sigma‐Aldrich, ≈13 ohm sq^−1^). The compact TiO_2_ was deposited by spray pyrolysis at 500 °C, using a solution of Titanium(IV) diisopropoxide bis(acetylacetonate) (Sigma‐Aldrich, 75%) diluted in absolute ethanol with a volume ratio of 1:19. Subsequently, the SbSeI absorber was grown in accordance with the methodology described above, using the corresponding synthesis conditions shown in Table 4. Finally, MoO_3_ and Au contacts were deposited by thermal evaporation. In order to outline the individual solar cells, Au deposition was carried out using a patterned mask featuring circular openings, each with an area of 0.07 cm^2^. Current–voltage (JV) measurements under 1 sun illumination were carried out with a Keithley 6430 source meter in a four‐point configuration, using an LED‐based G2V calibrated AAA solar simulator and light intensity of 87.8 mW cm^−2^. Measurements were performed in a superstrate configuration, that is, light incident through om the glass substrate.

Photocatalytic ethanol dehydrogenation. BiSeI films were prepared on glass substrates following the procedure shown above. The films were then mechanically removed to obtain BiSeI in powder form. The photocatalytic mixture was composed of commercial TiO_2_ P90 powder (Degussa, ≈90 m^2^ g^−1^), 1 wt.% BiSeI powder, and 1 wt.% gold nanoparticles (from Au(III) acetate, Alfa Aesar, 99.9%). To ensure homogeneous blending, the mixture was processed using a high‐speed vibrating ball mill (Pulverisette 23, Fritsch) for 10 min at a frequency of 15 Hz, with a ball‐to‐powder ratio of 45. For the catalyst application, 26.1 mg of the catalyst powder was dispersed in ethanol by ultrasonication and deposited onto the central part (4 cm diameter) of a circular cellulose membrane (F473 grade, 73 g m^−2^). The membrane was dried at 50 °C to evaporate the ethanol and stabilize the catalyst before reaction. It was then placed in the photoreactor so that the catalyst faced the UV–vis light source. The system was sealed to maintain a controlled environment for the photocatalytic reaction.

The photocatalytic tests were performed in a tubular fixed‐bed continuous flow photoreactor under heterogeneous solid–gas conditions at room temperature, 1 atm. The reactant stream, constituted of ethanol and deionized water in a 1:9 molar ratio (EtOH: H_2_O), was generated by bubbling an argon flow (20 mL min^−1^) through a Drechsel bottle containing the liquid mixture. Ethanol was included into the mixture to raise the VBM of the photocatalyst, enabling the oxidation reaction. The light source used UVA radiation with a total intensity of 700 W m^−^^2^. The photoreactor was connected to a gas chromatograph (Micro‐GC‐490‐PRO), allowing analysis of the reaction products every 4 min and providing real‐time monitoring throughout the experiment.

Three photocatalytic samples were tested individually: TiO_2_, TiO_2_ + 1 wt.% Au, and TiO_2_ + 1 wt.% Au + 1 wt.% BiSeI. A two‐step analysis was performed to evaluate the role of visible light in hydrogen (H_2_) and acetaldehyde (C_2_H_4_O) production:

Step 1: UV light was turned on until the production of H_2_ and C_2_H_4_O stabilized.
Step 2: Both UV and visible light were applied until a new stable level was reached.

PEC prototypes were prepared using a thin film superstrate configuration based on the following structure: glass/TiO_2_/SbSeI. First, TiO_2_ was deposited by spray‐pyrolysis, followed by SbSeI synthesis – in accordance with the methodology described above in greater detail. Then, the samples were scratched on one edge to expose the FTO substrate. Following this, the FTO‐exposed surface was soldered to a Cu wire with In, and the assembly was protected with a transparent epoxy resin, guaranteeing that no scratched regions would be exposed to the electrolyte. PEC performance was evaluated using an Autolab workstation in a conventional 3‐electrode configuration. A Pt foil was used as the counter electrode, Ag/AgCl in saturated KCl was used as the reference electrode, and the glass/TiO_2_/SbSeI device acted as the working electrode. A 0.1 C electrolyte solution was used (at pH = 7), based on a mixture of monobasic dihydrogen phosphate (KH_2_PO_4_) and dibasic monohydrogen phosphate (K_2_HPO_4_).

Linear sweep voltammetry (LSV) was performed under simulated sunlight provided by a solar simulator. All measurements were conducted under standard 1 Sun AM 1.5 conditions, using an anodic scan direction at a scan rate of 0.01 V s^−1^. Each device was tested sequentially under chopped light, continuous light, and dark conditions. Moreover, three consecutive rounds of continuous light exposure were performed to evaluate the stability of the devices.

### X‐Ray Diffraction

XRD patterns were obtained with a Bruker D8 Advance equipment in Bragg–Brentano configuration, using CuKα (*λ* = 1.54187 Å) radiation and 2*θ* range from 10° to 80°, with step size 0.02°. The patterns were analyzed with X'Pert HighScore software. Pattern matching refinement was performed using the FullProf suite.

### Scanning Electron Microscopy and Energy Dispersive X‐Ray Spectroscopy

SEM micrographs were acquired in top‐view and cross‐section conformation using a Zeiss Series Auriga field‐emission microscope, with an acceleration voltage of 5 kV and working distance ranging between 3 to 5 mm. EDX top‐view mappings were performed for compositional analysis, using the AZtec software from Oxford Instruments for data analysis.

### Transmission Electron Microscopy

TEM characterization was carried out using a FEI Talos F200X field emission operating at 200 keV. EDX were performed using four symmetrical EDS detectors.

Due to the characteristics of the materials, sample preparation was specifically adapted; that is, films presenting a clear needle‐like morphology were scraped meticulously to peel‐off single crystal structures, which were then deposited onto the TEM sample holder for analysis. This procedure was applied to SbSI, SbSeI, and all Bi chalcohalides. On the other hand, SbSBr and SbSeBr, which exhibited compact structures, were processed by focused ion beam to obtain ultra‐thin lamellae. Of all the materials, only BiSeI has presented difficulties to isolate single crystals for TEM‐EDP analysis; as a result, it was not possible to carry out the measurements in this particular case. See Figure S15 (Supporting Information) for the corresponding TEM images of the lamellae and needles for sample preparation.

### Transmission UV–Vis Spectroscopy and Photothermal Deflection Spectroscopy

Transverse PDS was used in this work for optical analysis (bandgap and absorption coefficient). The setup consisted of a 100 W tungsten halogen lamp, PTI 01–0002 monochromator (two‐grating monochromator with spectral range of 400–2000 nm), and Thorlabs MC1000 optical chopper (4 Hz light modulation frequency). A Signal Recovery 7265 lock‐in amplifier was connected to a Hamamatsu C10442‐02 PSD position sensitive detector to measure the deflection of the MC6320C laser probe beam (10 mW). For measurements, samples were located in a quartz cell filled with Fluorinert TM FC‐40 liquid. A personal computer controlled the monochromator, changed the order filters, and stored the PDS signal read from the lock‐in amplifier. Additionally, optical analyses were also performed by transmission UV–vis spectroscopy, using a Shimadzu UV‐3600 equipment with UVprobe software which allowed the spectral and photometric analysis of solid and liquid samples, with a total wavelength range of 185–3300 nm and resolution of 0.1 nm. The instrument was equipped with three detectors: PMT, InGaAs, and PbS. The bandgap was obtained using the Tauc model, and the first‐derivative method of the transmittance spectra.

### X‐Ray and UV Photoelectron Spectroscopy

XPS and UPS experiments were performed in ESFOSCAN at CCiTUB, an equipment based on the PHI VersaProbe 4 instrument from Physical Electronics (ULVAC‐PHI). XPS measurements were done with a monochromatic focused X‐ray source (Aluminium Kα line of 1486.6 eV) calibrated using the 3d5/2 line of Ag with a full width at half maximum (FWHM) of 0.6 eV. The analysed area was a circle of 100 µm of diameter, sample placed at 45° with respect to the analyzer axis, and the selected resolution for the spectra was 224 eV of Pass Energy and 0.8 eV step^−1^ for the general spectra, and 27 eV of Pass Energy and 0.1 eV step^−1^ for the high‐resolution spectra of the selected elements. A combination of low‐energy electrons (less than 5 eV) and low‐energy Ar^+^ ions (less than 100 eV) was used in order to discharge samples if necessary. Measurements were referenced to the C 1s signal, whose binding energy was equal to 284.8 eV in adventitious carbon (from atmospheric contamination). On the other hand, UPS measurements were done using a Helium source (He I line of 21, 22 eV) calibrated using an Ag sample in which the work function (WF) calculated was of 4, 27 eV. The analysed area was a spot of ≈1.5 mm of diameter, the sample placed at 90° with respect to the analyser axis, and the selected resolution for the spectra was 1.3 eV of Pass Energy and 0.01 eV step^−1^. Measurements were done with and without a bias of ≈10 eV, in order to create a well‐defined onset of secondary electrons for ionization energy (IE) and work function (WF) calculation purposes. Both XPS and UPS measurements were made in an ultra‐high vacuum (UHV) chamber at a pressure between 5 × 10^−10^ and 5 × 10^−9 ^Torr. Moreover, in order to measure beyond the Carbon (adventitious) contaminated surface, sputtering with a monoatomic Ar^+^ ion gun (at 0.5 keV) was done, with no damage appreciated in the samples when looking at the high‐resolution obtained spectra. The analysis and fitting of the spectra were carried out with the Multipak V. 9.0.8 program.

### Raman Spectroscopy

Raman spectroscopy measurements were carried out with a Renishaw inVia Qontor instrument, with an excitation wavelength of 532 nm and nominal 100 mW output power, coupled with 2400 lines mm^−1^ visible grating and a Leica DM2700 microscope (with 100× objective) for confocal measurements. For data collection, between 0.5% to 5% of the nominal power was used. 5 accumulations with 20 s of exposure time for each accumulation (total exposure time: 100 s per measurement) over 5 different areas of each sample were performed on each compound. Laser power conditions were selected based on a power study that measured the Raman spectrum at the same point on the material with increasing laser power densities, starting from the lowest power available. For each laser power, the spectrum was monitored for changes in peak positions, peak widths, or the appearance of new peaks. The highest power for which no changes in these parameters were observed was taken as the optimal laser power for measurements.

### Photoluminescence

PL measurements were carried out with a WITec Alpha 300 R confocal Raman microscope. Measurements were performed using a 488 nm laser coupled with a 150 lines mm^−1^ visible grating and a 100× magnification objective for analysis. For data collection, a single line scan accumulation of 6 points using 0.5 mW as incident power and an integration time of 10 s per point was carried out on different samples.

## Conflict of Interest

The authors declare no conflict of interest.

## Supporting information



Supporting Information

## Data Availability

The data that support the findings of this study are available from the corresponding author upon reasonable request.
