# Supplementary material for: Ribbons of Light: Emerging (Sb,Bi)(S,Se)(Br,I) Van der Waals Chalcohalides for Next‐Generation Energy Applications
Source: Small. 2025 Jul 23;21(37):e05430. doi: 10.1002/smll.202505430 (PMC12444836; doi:10.1002/smll.202505430)
Supplement: Supplementary file 1 — Supporting Information [file SMLL-21-e05430-s001.pdf]

NANO · MICRO
small

Supporting Information

for *Small*, DOI 10.1002/smll.202505430

Ribbons of Light: Emerging (Sb,Bi)(S,Se)(Br,I) Van der Waals Chalcohalides for Next-Generation Energy Applications

Ivan Caño, Alejandro Navarro-Güell, Edoardo Maggi, Axel Gon Medaille, David Rovira, Alex Jimenez-Arguijo, Oriol Segura, Arnau Torrens, Maykel Jimenez, Cibrán López, Pol Benítez, Claudi Cazorla, Zac Jehl, Yuancai Gong, José-Miguel Asensi, Lorenzo Calvo-Barrio, Lluís Soler, Jordi Llorca, Josep-Lluís Tamarit, Beatriz Galiana, Mirjana Dimitrievska, Nazaret Ruiz-Marín, Hao Zhe Chun, Lydia Wong, Joaquim Puigdollers, Marcel Placidi and Edgardo Saucedo*

Supporting Information

1. Structural and vibrational properties

The eight chalcohalide parent compounds were found to present a very similar structure, being all the lattice vectors in excellent agreement with the experimental results (Table 1). All lattice angles (α, β, γ) where found to be equal to 90°.

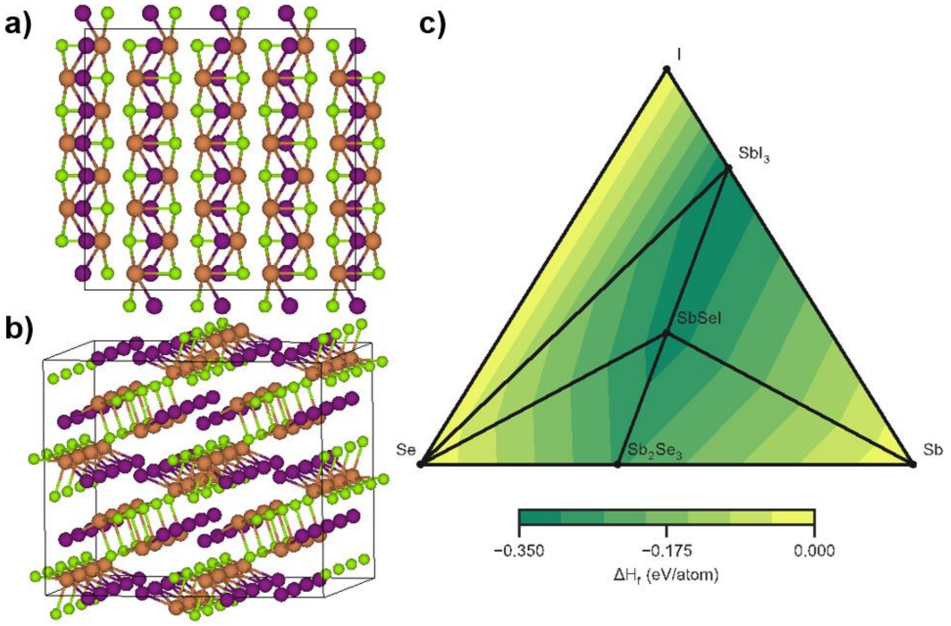

Figure S1. Chalcohalide compounds general crystal structure particularized for SbSeI (Sb, Se and I atoms are represented with brown, green, and purple coloured spheres). The orthorhombic Pnma structure **a)** along the [001] direction and **b)** in a three-dimensional view **c)** Calculated convex-hull surface for SbSeI as an illustrative example

Table S1. Lattice vectors from first-principles calculation (at PBEs+D3 level of theory) and from experimental diffraction

**Element**	a^{DFT} (Å)	b^{DFT} (Å)	c^{DFT} (Å)	a^{exp} (Å)	b^{exp} (Å)	c^{exp} (Å)
**BiSBr**	4.03	8.06	9.52			
**BiSeBr**	4.08	8.14	10.12	4.11	8.19	10.46
**BiSI**	4.14	8.34	10.02			
**BiSeI**	4.19	8.55	10.34	4.22	8.71	10.58
**SbSBr**	3.90	8.05	9.58	3.97	8.24	9.75
**SbSeBr**	3.97	8.14	10.09	4.02	8.32	10.23
**SbSI**	4.04	8.31	9.97	4.11	8.57	10.19
**SbSeI**	4.10	8.51	10.27	4.15	8.69	10.39

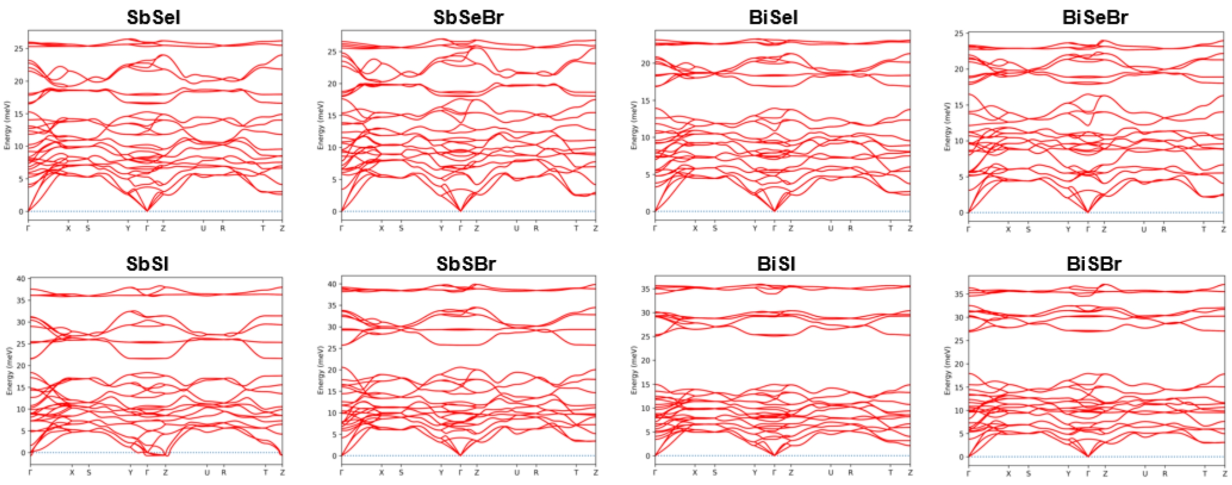

Figure S2: Vibrational phonon spectra along high-symmetry **k**-path.

A second orthorhombic *Pnma* phase was found to be energetically competitive with respect to the orthorhombic ground state (see **Figure S1**). This metastable structure consists on the vertical dislocation of a [001] plane (see **Figure S3**).

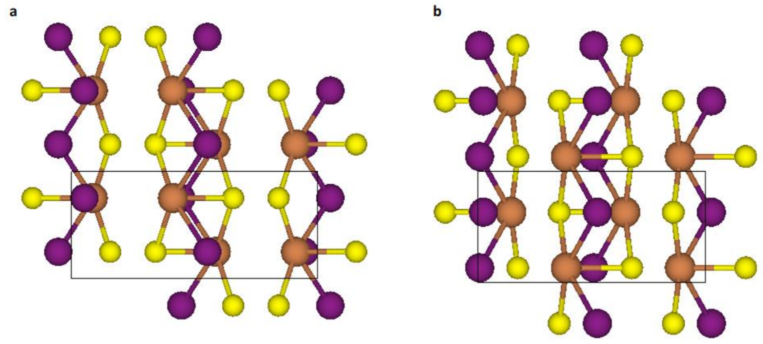

Figure S3. Comparison for SbSI between **a)** Pnma phase found for all chalcohalide parent compounds and **b)** the competitive Pnma structure. This new phase consists on a vertical dislocation of a [001] plane by half the unit cell. Sb, S and I atoms are represented with brown, green, and purple coloured spheres. The unit cell is depicted with a rectangle of black, solid lines

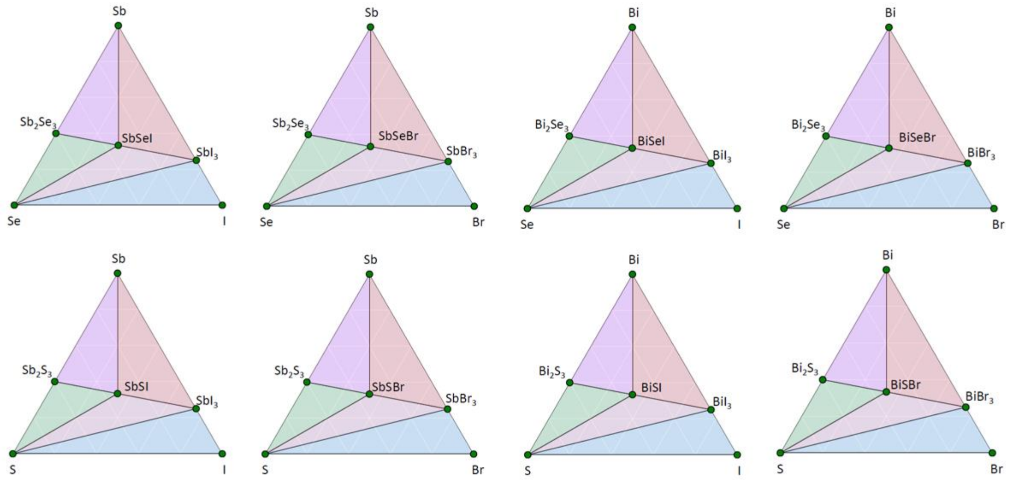

Figure S4. Calculated convex-hull surface of the eight chalcohalide compounds. Green points indicate stable structures

Experimental Raman active frequencies and corresponding Γ-phonon frequencies from first-principles calculations are reported in **Table S2**, along with the theoretically calculated contribution of each element to the vibration.

Table S2. Experimental Raman-active frequencies and corresponding first-principles *Γ*-phonon frequencies for each chalcohalide parent compound. It is as well include de contribution of each element to the specifc vibration

Element	f^{exp} (cm^{-1})	f^{DFT} (cm^{-1})	Bi/Sb (%)	S/Se (%)	Br/I (%)
**BiSBr**	89	87	61.62	16.08	22.30
	106	107	64.48	14.81	20.71
	119	116	36.27	7.52	56.21
	124	125	27.26	5.71	67.03
	241	243	13.05	84.30	2.65
	244	245	13.78	82.44	3.78
	285	284	16.57	78.54	4.89
**BiSeBr**	78	79	30.22	10.55	59.23
	88	87	8.69	2.95	88.36
	91	901	54.84	24.52	20.64
	102	98	29.83	23.25	46.92
	109	111	29.43	12.81	57.76
	132	133	27.60	18.3	54.1
	140	144	24.53	60.13	15.34
	168	169	28.78	64.42	6.80
	181	184	29.69	67.03	3.28
**BiSI**	86	86	56.74	14.26	29.0
	107	109	36.65	9.04	54.31
	224	235	22.93	70.18	6.89
	282	282	23.62	72.16	4.22
	285	284	17.07	79.24	3.69
**BiSeI**	74	74	17.63	0.72	81.65
	84	85	52.67	23.34	23.99
	94	95	31.46	17.15	51.39
	108	102	27.21	15.67	57.12
	114	112	29.47	20.38	50.15
	134	136	22.00	59.39	18.61
	151	148	21.86	73.10	5.04
	180	181	31.11	55.28	13.61
**SbSBr**	77	80	24.37	8.20	67.43
	117	116	47.94	16.21	35.85
	148	143	41.66	15.83	42.51
	229	236	10.19	83.7	6.11
	265	264	28.57	64.59	6.84
	320	316	24.67	73.64	1.69
**SbSeBr**	96	93	18.51	4.20	77.29
	102	99	44.92	30.34	24.74
	124	124	25.04	23.36	51.60
	147	149	21.43	68.90	9.67

	151	151	23.43	67.30	9.27
	167	183	37.18	56.61	6.21
	190	189	37.63	54.18	8.19
	210	211	39.14	55.71	5.15
**SbSI**	74	72	36.03	9.00	54.97
	107	109	44.00	12.47	43.53
	139	145	48.67	20.84	30.49
	231	229	9.33	86.35	4.32
	249	250	28.31	66.59	5.10
	319	315	24.62	74.10	1.28
**SbSeI**	93	96	41.98	25.57	32.45
	113	113	31.70	29.42	38.88
	135	135	27.88	58.38	13.74
	165	174	39.56	54.53	5.91
	179	178	41.19	55.11	3.70
	206	208	40.45	48.78	10.77

2. Thermodynamic stability

All chalcohalides lie below the convex-hull surface (**Table S3**), thus they can be confidently assumed to be thermodynamically stable at temperatures near ambient.

Table S3: Energies above the convex-hull for the chalcohalide parents (first row), with the corresponding formation energies of the these elements (second row) and their secondary phases (third row). Energies are given in eV/atom

ΔH_c^{BiSBr}	ΔH_c^{BiSeBr}	ΔH_c^{BiSI}	ΔH_c^{BiSeI}	ΔH_c^{SbSBr}	ΔH_c^{SbSeBr}	ΔH_c^{SbSI}	ΔH_c^{SbSeI}
-0.02	-0.01	-0.01	-0.01	-0.05	-0.05	-0.03	-0.03

ΔH_f^{BiSBr}	ΔH_f^{BiSeBr}	ΔH_f^{BiSI}	ΔH_f^{BiSeI}	ΔH_f^{SbSBr}	ΔH_f^{SbSeBr}	ΔH_f^{SbSI}	ΔH_f^{SbSeI}
-0.55	-0.54	-0.43	-0.42	-0.46	-0.43	-0.35	-0.33

ΔH_f^{Bi2S3}	ΔH_f^{Bi2Se3}	ΔH_f^{BiI3}	ΔH_f^{BiBr3}	ΔH_f^{Sb2S3}	ΔH_f^{Sb2Se3}	ΔH_f^{SbI3}	ΔH_f^{SbBr3}
-0.39	-0.38	-0.47	-0.72	-0.29	-0.26	-0.35	-0.54

where ΔH_c is the energy above the convex-hull and $\Delta \mathrm{H_f}$ is the formation energy of the corresponding element (indicated with an upper index).

3. Optoelectronic properties

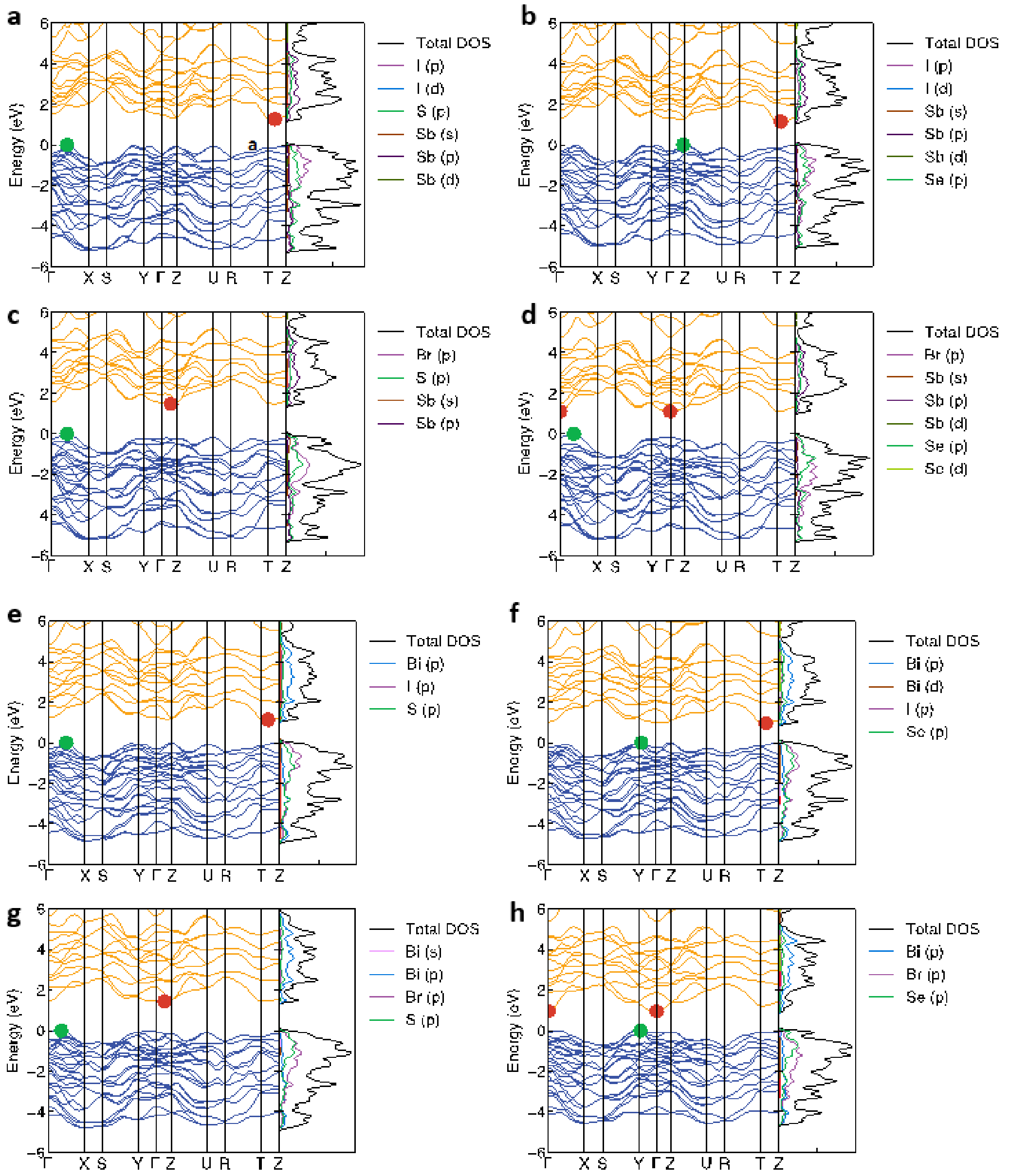

Figure S5. Energy-momentum bands structures for **a)** SbSI, **b)** SbSeI, **c)** SbSBr, **d)** SbSeBr, **e)** BiSI, **f)** BiSeI, **g)** BiSBr and **h)** BiSeBr along high-symmetry **k**-paths in the Brillouin zone, computed at PBEsol+SOC level of theory. Conduction and valence bands are represented in yellow and blue colours, respectively, and the contribution of each type of orbital to them are shown in the corresponding density of states (the Fermi energy level is set to 0 eV). Green and red dots represent the top of the valence band and the bottom of the conduction band, respectively. The exact same trends analysed in the main text are conserved

Similar trends are found for all energy-momentum bands structures of the eight chalcohalide parent compounds (**Figures S5** and **S6**).

To demonstrate the influence of the different correction schemes to the computation of the band gap, the same calculations have been performed for the range-separated hybrid HSE06 functional with spin-orbit, van der Waals and no corrections (**Table S4**). It is confidently proved the relevance of spin-orbit corrections for these materials, achieving with this scheme much higher agreement with the experiments.

Table S4. Comparison of band gaps resulting from different levels of theory (HSE06+SOC, HSE06+D3 and HSE06). Remarkable differences are achieved with the introduction of SOC corrections, which are in close agreement with experiments.

**Element**	$E_g^{(HSE06+SOC)}$ (eV)	$E_g^{(HSE06+D3)}$ (eV)	$E_g^{(HSE06)}$ (eV)
**BiSBr**	1.84	2.33	2.40
**BiSeBr**	1.30	1.86	2.01
**BiSI**	1.49	2.16	2.19
**BiSeI**	1.25	1.91	1.92
**SbSBr**	1.93	2.05	2.14
**SbSeBr**	1.45	1.59	1.71
**SbSI**	1.70	1.88	1.93
**SbSeI**	1.50	1.70	1.71

Energy loss, extinction coefficient, reflectivity and refractive index are also computed for the chalcohalides (**Figures S6** to **S9**). Similar trends to those depicted in the Main Text for the absorption coefficient are found for these optical properties as well.

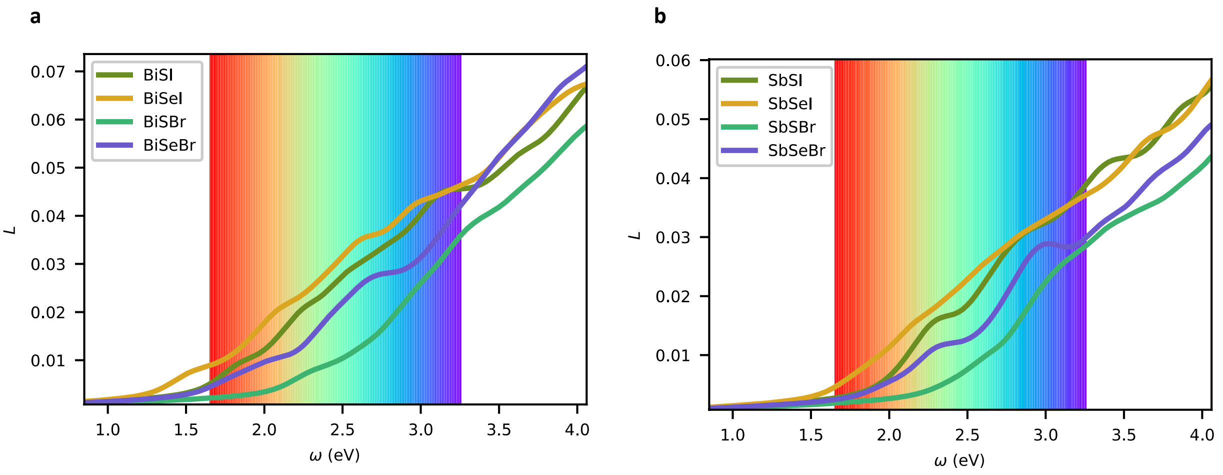

Figure S6. Energy loss calculated (at 0K) for **a)** Bi and **b)** Sb based chalcohalides, computed at HSE06+SOC level of theory. Red and blue vertical lines denote the infrared and ultraviolet limits of the visible zone, respectively

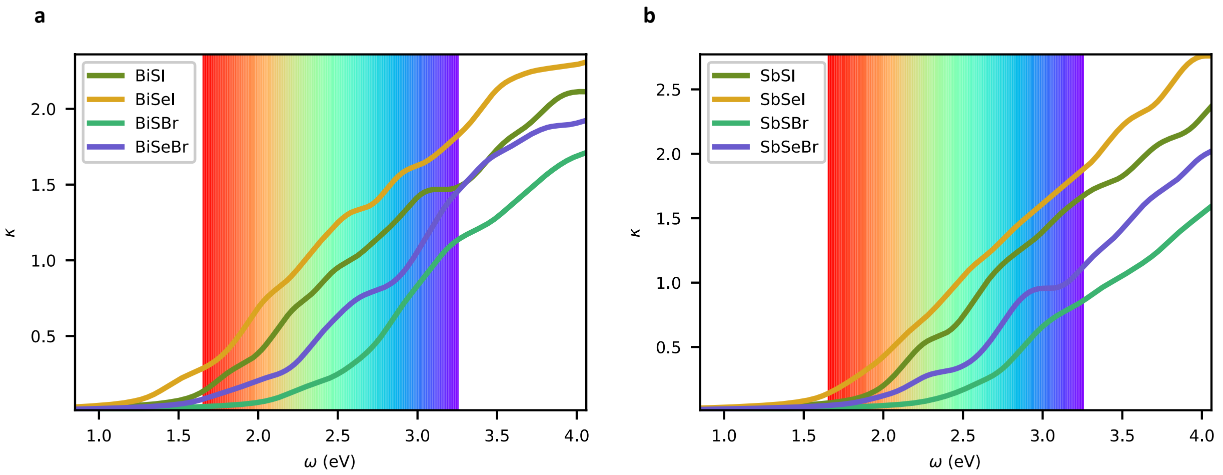

Figure S7. Extinction coefficient calculated (at 0K) for **a)** Bi and **b)** Sb based chalcohalides, computed at HSE06+SOC level of theory. Red and blue vertical lines denote the infrared and ultraviolet limits of the visible zone, respectively

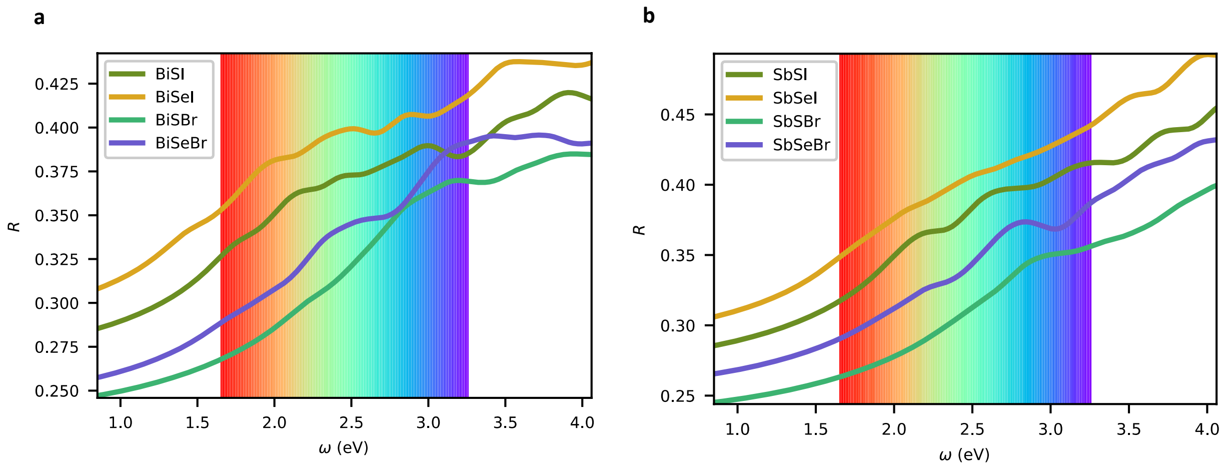

Figure S8. Reflectivity calculated (at 0K) for **a)** Bi and **b)** Sb based chalcohalides, computed at HSE06+SOC level of theory. Red and blue vertical lines denote the infrared and ultraviolet limits of the visible zone, respectively

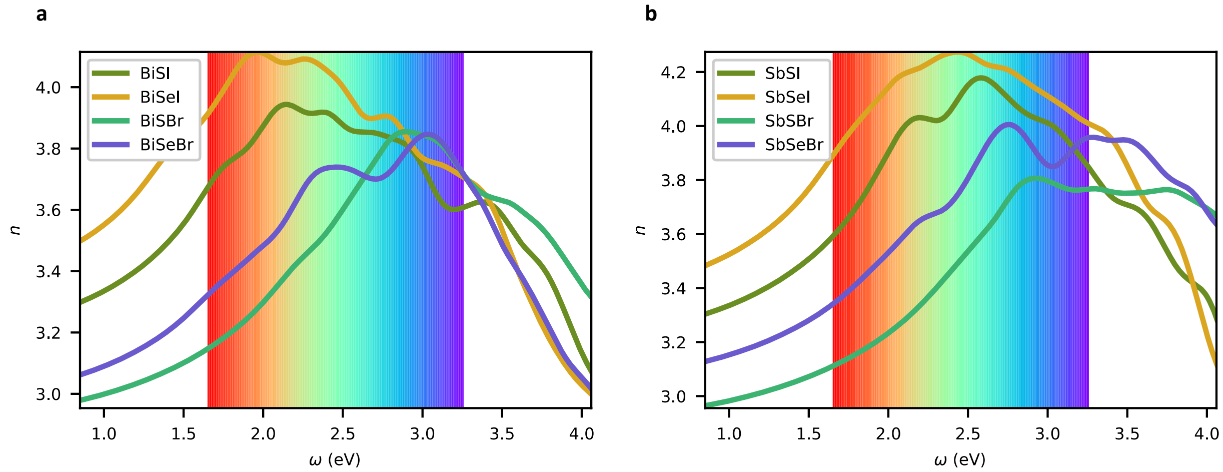

Figure S9. Refractive index calculated (at 0K) for a) Bi and b) Sb based chalcohalides, computed at HSE06+SOC level of theory. Red and blue vertical lines denote the infrared and ultraviolet limits of the visible zone, respectively

4. Band alignments

Band alignments were also computed at PBEs level of theory (**Table S5**). The VBM was computed with PBEs functionals, and then the CBM was extrapolated with the bandgap at HSE06+SOC level of theory (which has been proved more accurate). Consequently, these results are in some agreement with the HSE06+SOC band alignments (Main text **Figure 6**), as PBEs functional was only used to compute energies from non-excited states.

Table S5. Top of the valence band, computed at the PBEs level of theory, and bottom of the conduction band, extrapolated with the HSE05+SOC band gaps, for the chalcohalide parent materials

**Element**	CBM (eV)	VBM (eV)
**BiSBr**	-4.37	-6.21
**BiSeBr**	-5.50	-6.81
**BiSI**	-6.28	-7.77
**BiSeI**	-5.04	-6.29
**SbSBr**	-4.75	-6.68
**SbSeBr**	-4.85	-6.29
**SbSI**	-5.55	-7.24
**SbSeI**	-5.30	-6.80

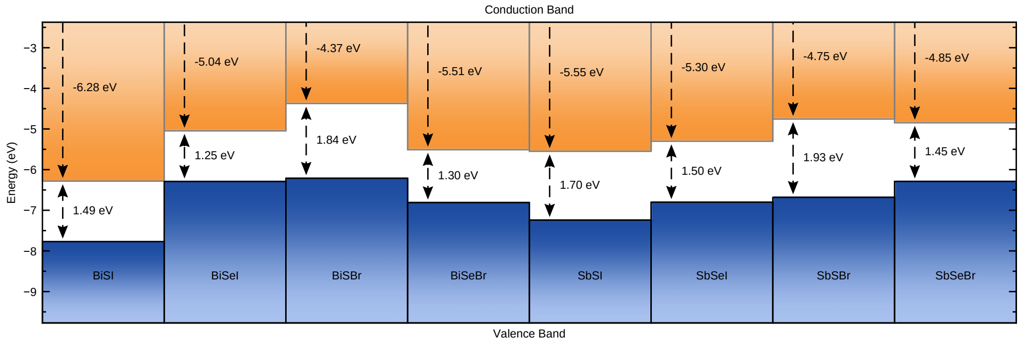

Figure S10. Top of the valence band (blue) and bottom of the conduction (orange) band for the chalcohalide materials family computed at the PBEs level of theory. The corresponding energy band gaps are also indicated. The wide range covered by the band alignments suggest promising energy applications for the chalcohalide materials family

4. XRD Analysis

XRD analysis by LeBail refinement have been performed using the FullProf suite.

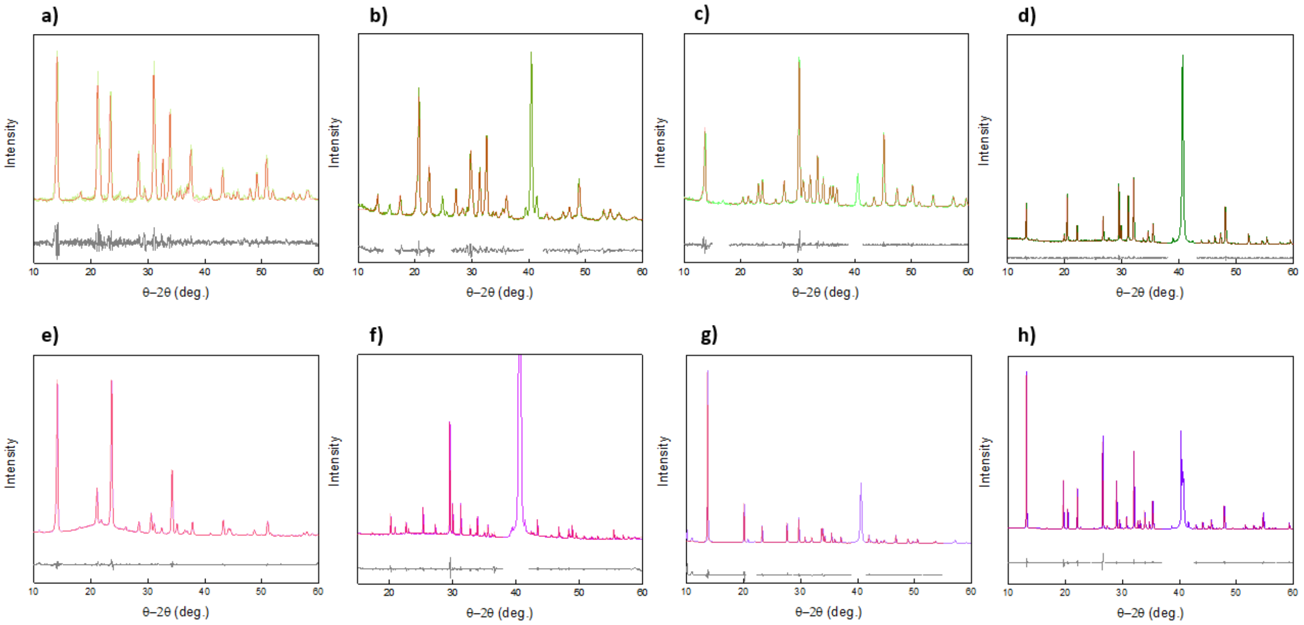

Figure S11. Experimental (coloured) and Le Bail (red) refined patterns, along with the difference profile (grey curve), of a series of MChX compounds comprising: **a)** SbSBr, **b)** SbSI, **c)** SbSeBr, **d)** SbSeI, **e)** BiSBr, **f)** BiSI, **g)** BiSeBr, **h)** BiSeI

<u>OCD sheets used in Figure 7</u>:

BiSBr → OCD-1535795
BiSeBr → ICSD-76649
BiSeI → OCD-2010577
BiSI → OCD-1535800
SbSBr → OCD-1521208
SbSeBr → cif not available
SbSeI → OCD-1008204
SbSI → OCD-1537520
Sb2Se3 → OCD-2310974
Sb2S3 → OCD-9003459

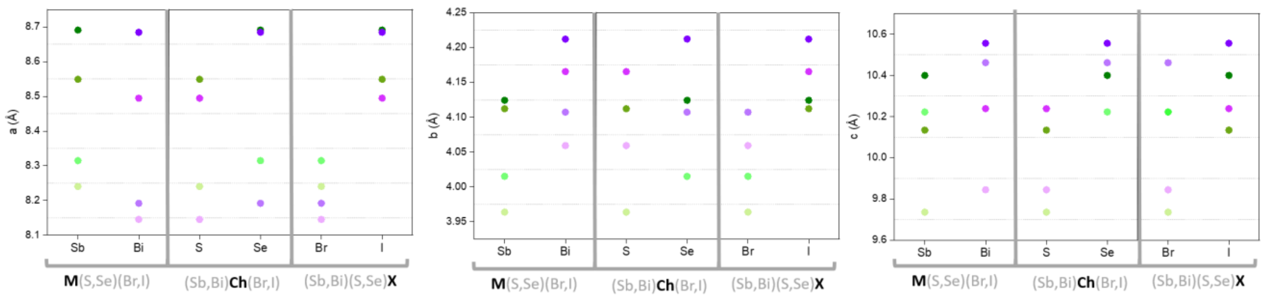

Figure S12. Individual lattice parameters of the chalcohalide compounds, grouped by element substitution type: metal M, chalcogen Ch, and halide X

Table S6. Cell parameters and cell volume

Element	a (A)	b (A)	c (A)	V (A^3)
**BiSBr**	8.145	4.059	9.845	325.5
**BiSeBr**	8.192	4.107	10.461	352.0
**BiSI**	8.495	4.165	10.239	362.3
**BiSeI**	8.685	4.212	10.556	386.2
**SbSBr**	8.240	3.964	9.736	318.0
**SbSeBr**	8.321	4.017	10.230	341.9
**SbSI**	8.549	4.112	10.134	356.2
**SbSeI**	8.681	4.124	10.400	372.8

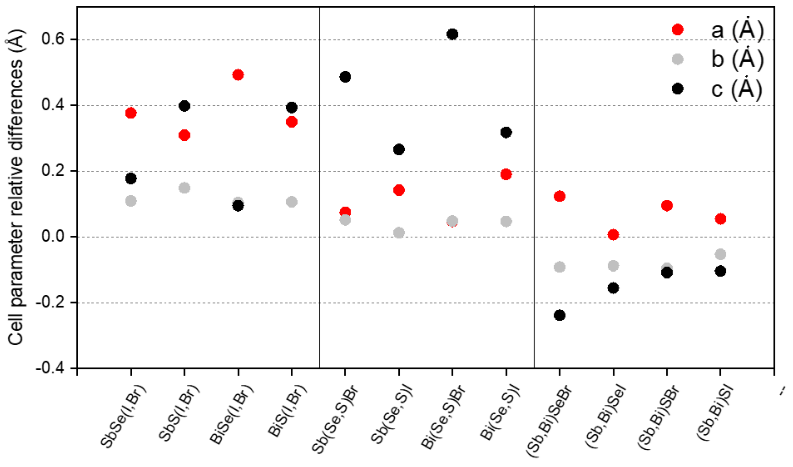

Figure S13. Cell parameter relative differences (determined by Le Bail refinement) of MChX compounds varying the halide (I,Br), chalcogenide (Se,S), and metal (Sb,Bi)

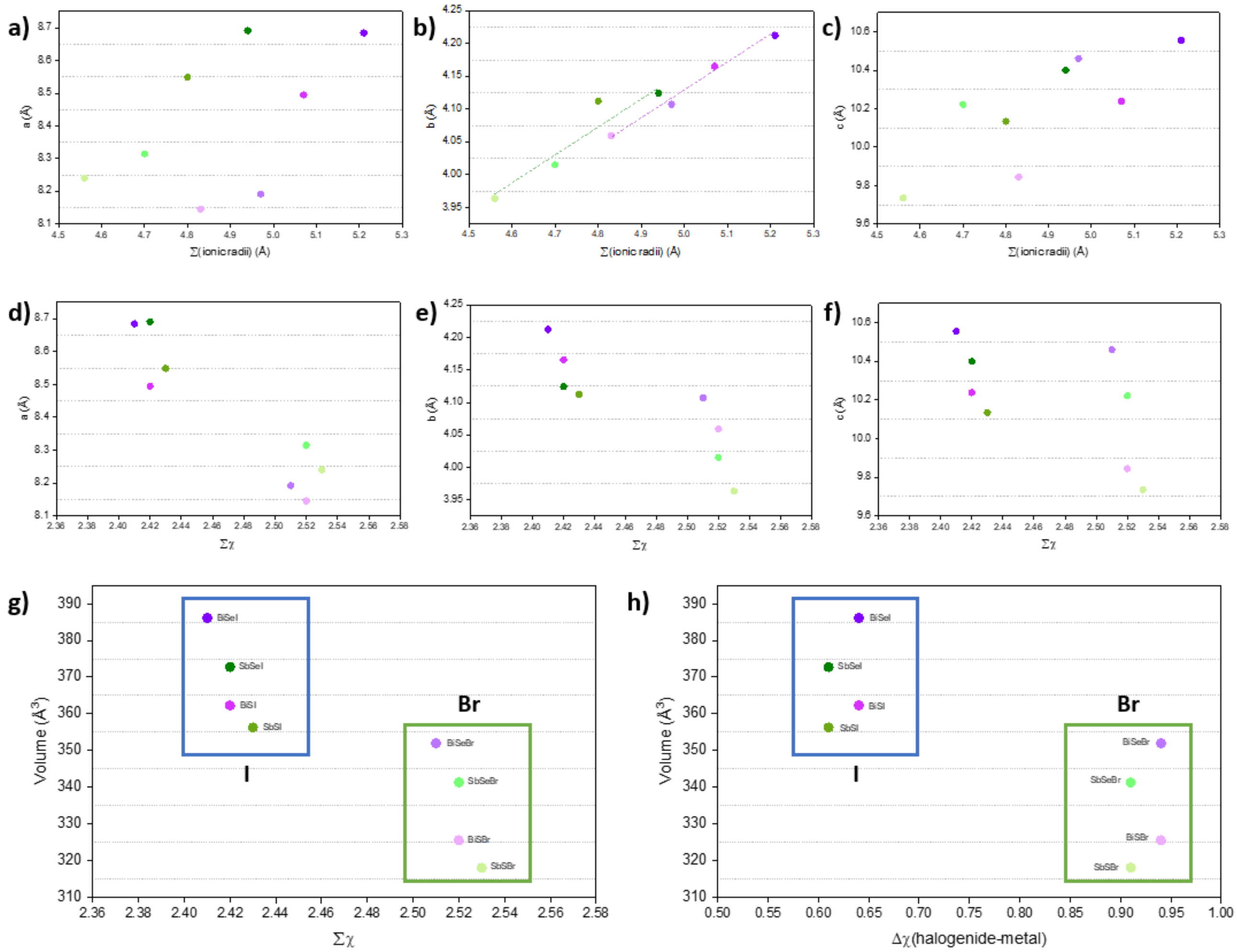

Figure S14. **a-c)** Cell parameters of MChX compounds as a function of the ionic radii sum of the constituent elements. **d-f)** Cell parameters of MChX compounds as a function of the electronegativity sum of the constituent elements. **g)** Cell volume of MChX compounds as a function of the electronegativity sum of the constituent elements. **h)** Cell volume of MChX compounds as a function of electronegativity difference between the halogen and meta

5. TEM Analysis

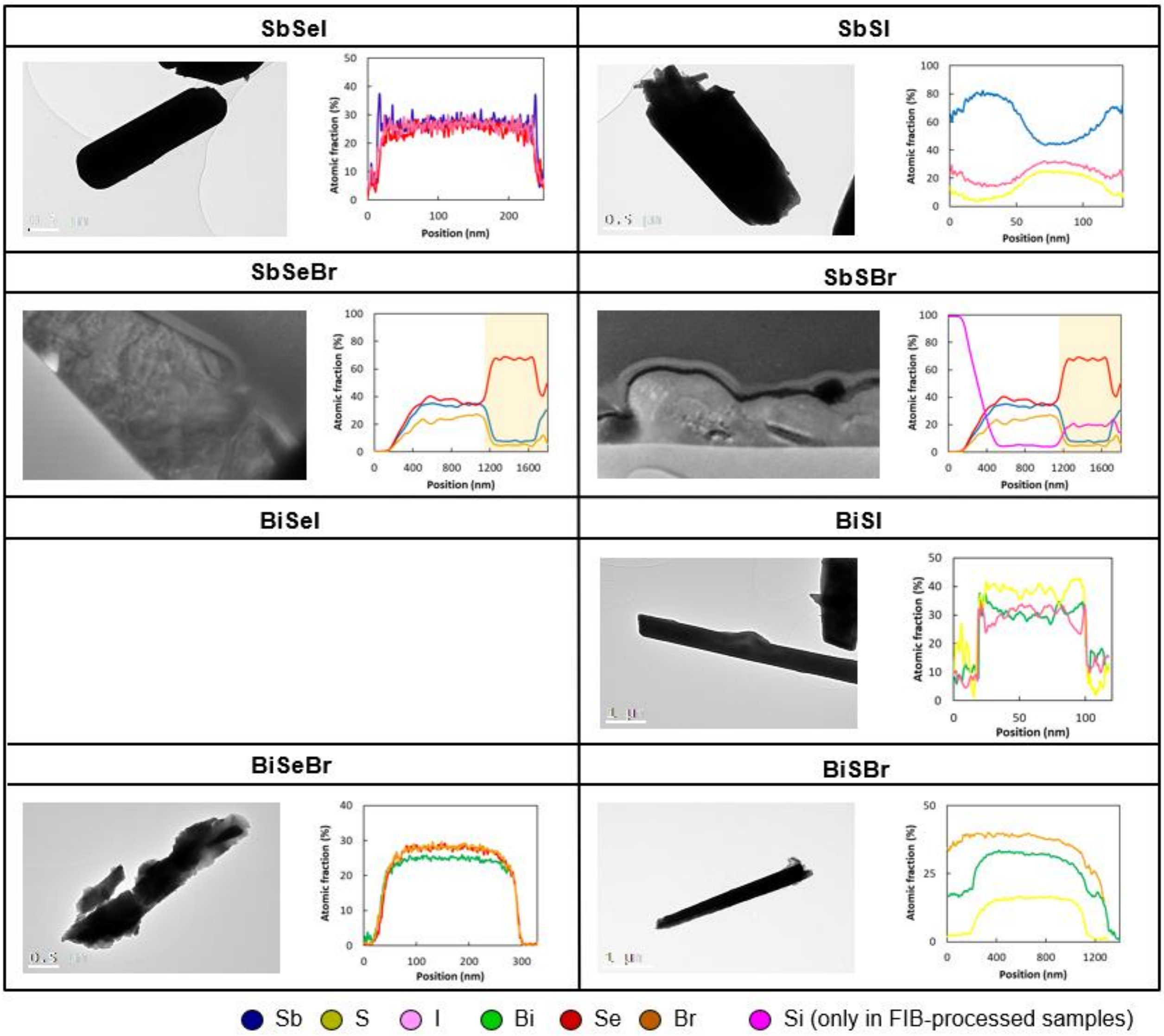

Figure S15. TEM images of lamellae and crystals used to perform the EDP and EDX analysis

6. Raman and PL analysis

Table S7. List of Raman peaks and fitted FWHM, extracted from the spectra in **Figure 14**. Literature Raman peaks are also included, except for BiSBr and SbSeBr, for which no complete Raman spectra have been reported so far (NRSR – No Raman Spectra Reported). Legend of the comments: SMO – Several Modes Overlapped / VLIP – Very Low Intensity Peak

	Exp. Raman freq. (cm^{-1})	FWHM	Lit. Raman freq. (cm^{-1})	Comments
SbSBr [1,2]	78	9.4	77	
	119	9.9	117	
	149	11.4	148	
	169		174	SMO
	231		217	SMO
	269			SMO
	321	5.7	322	
SbSI[3,4]	75	9.7		
	109	13.2	108	
	139	11.2	140	
	150		149	SMO
	211		218	SMO
	253		236	VLIP
	319	6.8	318	
SbSeBr - NRSR	74	13.2		
	101	4.8		
	126			SMO
	144			SMO
	210	5.6		
	254			VLIP
SbSeI[5]	71	7.5		
	93	5.0	90	
	114	9.5	110	
	135	13.5	130-140	
	165	8.4		
	179		183	SMO
	207	5.7	205	
	253			VLIP
BiSBr[6]	89		75(?)	VLIP
	119	9.8	121	
	148			SMO
	230		234	SMO
	242		250	SMO
	284	5.6	287	
BiSI[2,7,8]	79			SMO
	86	6.4	88	
	106	8.6	107	
	120		120	SMO
	221	17.0	220	
	267		272	VLIP / SMO
	284	7.0	282	
BiSeBr	79	5.5		

- NRSR	88			SMO
	110	15.6		
	120-150			SMO
	163			SMO
	168			SMO
	181	5.4		
	256			VLIP
BiSeI[2,9,10]	75	5.2	76	
	83		83	SMO
	95		97	SMO
	109		108	SMO
	120-140		139	SMO
	152	6.8	155	
	180	8.2	182	
	255	21.3		VLIP

Table S8. Maximum of the PL spectra (see **Figure 12**b of the main text)

**Material**	Maximum of Pl peak (eV)
**BiSBr**	1.99
**BiSeBr**	1.54
**BiSI**	1.64
**BiSeI**	1.38
**SbSBr**	1.81
**SbSeBr**	2.08
**SbSI**	1.91
**SbSeI**	1.74

7. XPS Analysis

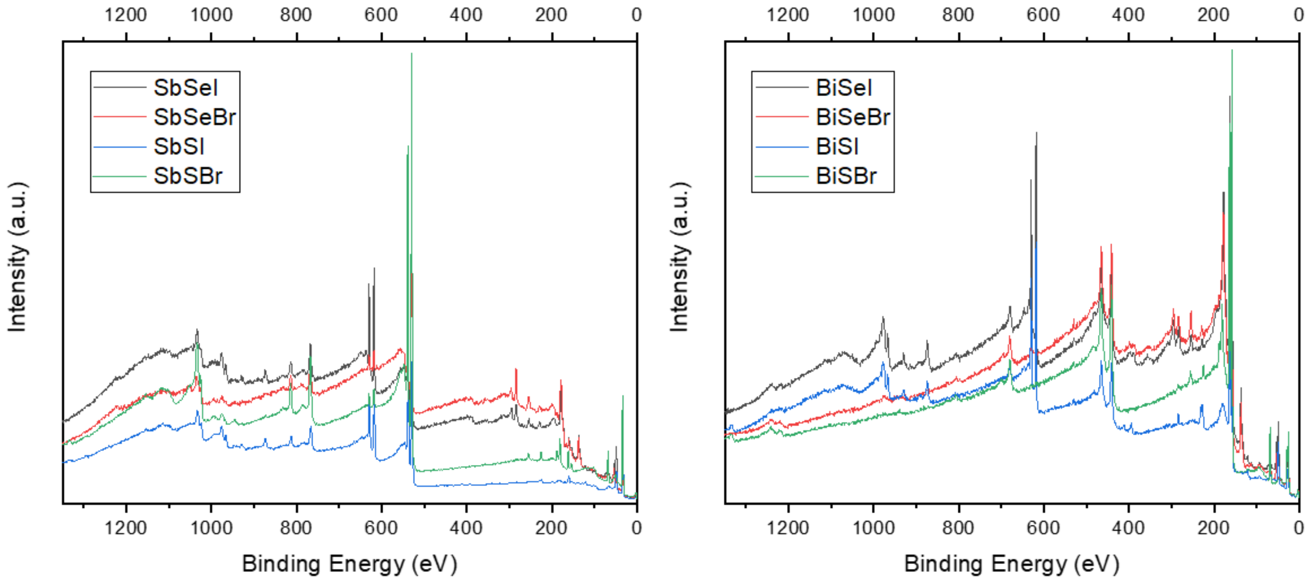

Figure S16. XPS spectra of Sb and Bi-based chalcohalides

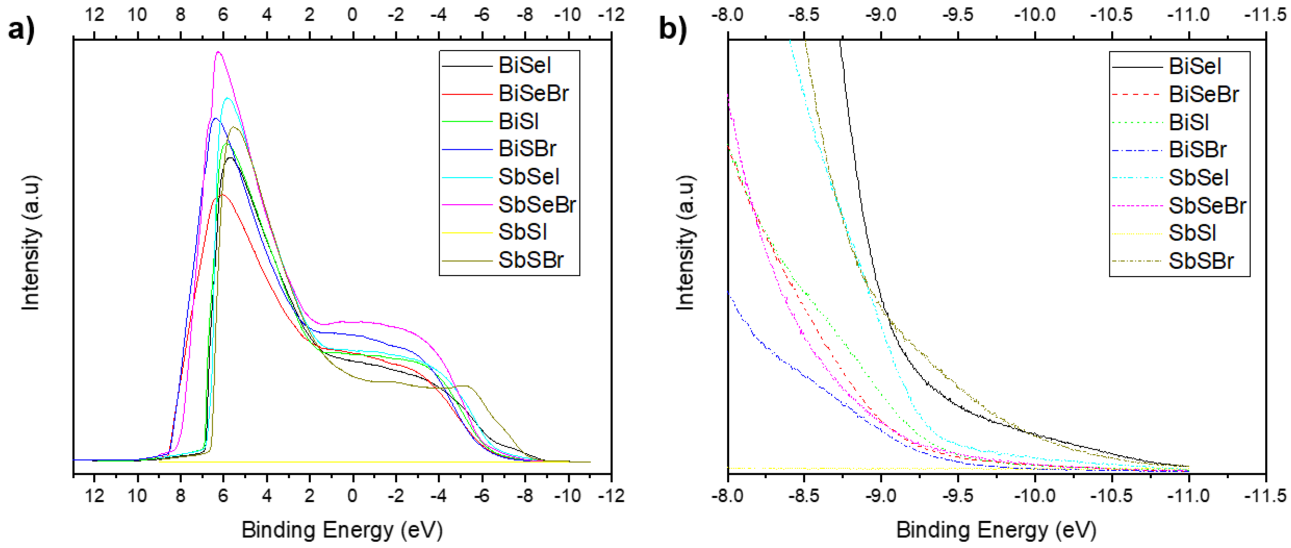

Figure S17. **a)** UPS measurements of Sb and Bi-based chalcohalide with applied BIAS at the sample, and **b)** the VBM region enlarged

References

1 T. Inushima, K. Uchinokura, K. Sasahara and E. Matsuura, *Raman spectra and lattice dynamics in ferroelectric SbSBr*, vol. 26.

2 M. K. Teng, M. Balkanski, M. Massot and M. K. Ziolkiewicz, *Optical Phonon Analysis in the AVBVICVII Compounds 173 phys*, 1974, vol. 62.

3 A. K. Pathak, M. D. Prasad and S. K. Batabyal, *Applied Physics A*, 2019, **125**, 213.

4 R. A. Groom, A. Jacobs, M. Cepeda, R. Drummey and S. E. Latturner, *Inorg Chem*, 2017, **56**, 12362–12368.

5 G. Kanchana and D. Arivuoli, *Indian Journal of Engineering & Materials Sciences*, 2001, **8**, 373–376.

6 E. Furman, O. Brafman and J. Makovsky, *Phys Rev B*, 1976, **13**, 1703–1710.

7 H. Sun, G. Yang, J. Chen, C. Kirk and N. Robertson, *J Mater Chem C Mater*, 2020, **8**, 13253–13262.

8 J. Li, B. Wang, F. Liu, J. Liu, M. Jia, Y. Lai, J. Li and Y. Liu, *ECS Solid State Letters*, 2012, **1**, 31–33.

9 Y. Li, S. Wang, J. Hong, N. Zhang, X. Wei, T. Zhu, Y. Zhang, Z. Xu, K. Liu, M. Jiang and H. Xu, *Small*, DOI:10.1002/smll.202302623.

10 C. An, X. Du, X. Chen, Y. Zhou, M. Zhang, Y. Zhou, J. Zhou and Z. Yang, *Phys Rev B*, 2023, **107**, 134501.

11 M. K. Hossain, G. F. I. Toki, A. Kuddus, M. H. K. Rubel, M. M. Hossain, H. Bencherif, M. F. Rahman, M. R. Islam and M. Mushtaq, *Sci Rep*, DOI:10.1038/s41598-023-28506-2.

12 W. Septina, Gunawan, S. Ikeda, T. Harada, M. Higashi, R. Abe and M. Matsumura, *Journal of Physical Chemistry C*, 2015, **119**, 8576–8583.

13 A. Slassi, *RSC Adv*, 2022, **12**, 12068–12077.
